# CCR2 macrophage response determines the functional outcome following cardiomyocyte transplantation

**DOI:** 10.1186/s13073-023-01213-3

**Published:** 2023-08-10

**Authors:** Praveen Vasudevan, Markus Wolfien, Heiko Lemcke, Cajetan Immanuel Lang, Anna Skorska, Ralf Gaebel, Anne-Marie Galow, Dirk Koczan, Tobias Lindner, Wendy Bergmann, Brigitte Mueller-Hilke, Brigitte Vollmar, Bernd Joachim Krause, Olaf Wolkenhauer, Gustav Steinhoff, Robert David

**Affiliations:** 1 Department of Cardiac Surgery, Rostock University Medical Centre, 18057 Rostock, Germany; 2 Department of Life, Light and Matter, University of Rostock, Albert-Einstein-Str. 25, 18059 Rostock, Germany; 3 Rudolf-Zenker-Institute for Experimental Surgery, Rostock University Medical Centre, 18057 Rostock, Germany; 4 Department of Systems Biology and Bioinformatics, Institute of Computer Science, University of Rostock, 18057 Rostock, Germany; 5 Institute for Medical Informatics and Biometry, Faculty of Medicine Carl Gustav Carus, Technische Universität Dresden, 01307 Dresden, Germany; 6 Center for Scalable Data Analytics and Artificial Intelligence (ScaDS.AI), Dresden/Leipzig, Germany; 7 Department of Cardiology, Rostock University Medical Centre, 18057 Rostock, Germany; 8 Institute of Genome Biology, Research Institute for Farm Animal Biology (FBN), 18196 Dummerstorf, Germany; 9 Core Facility for Microarray Analysis, Institute for Immunology, Rostock University Medical Centre, 18057 Rostock, Germany; 10 Core Facility Multimodal Small Animal Imaging, Rostock University Medical Centre, 18057 Rostock, Germany; 11 Core Facility for Cell Sorting & Cell Analysis, Laboratory for Clinical Immunology, Rostock University Medical Centre, 18057 Rostock, Germany; 12 Department of Nuclear Medicine, Rostock University Medical Centre, 18057 Rostock, Germany; 13 Stellenbosch Institute of Advanced Study (STIAS), Wallenberg Research Centre at Stellenbosch University, Stellenbosch, 7602 South Africa

**Keywords:** Immunocompromised, Myocardial infarction, Machine learning, Macrophages, Single-cell, Cell therapy

## Abstract

**Background:**

The immune response is a crucial factor for mediating the benefit of cardiac cell therapies. Our previous research showed that cardiomyocyte transplantation alters the cardiac immune response and, when combined with short-term pharmacological CCR2 inhibition, resulted in diminished functional benefit. However, the specific role of innate immune cells, especially CCR2 macrophages on the outcome of cardiomyocyte transplantation, is unclear.

**Methods:**

We compared the cellular, molecular, and functional outcome following cardiomyocyte transplantation in wildtype and T cell- and B cell-deficient Rag2^del^ mice. The cardiac inflammatory response was assessed using flow cytometry. Gene expression profile was assessed using single-cell and bulk RNA sequencing. Cardiac function and morphology were determined using magnetic resonance tomography and immunohistochemistry respectively.

**Results:**

Compared to wildtype mice, Rag2^del^ mice show an increased innate immune response at steady state and disparate macrophage response after MI. Subsequent single-cell analyses after MI showed differences in macrophage development and a lower prevalence of CCR2 expressing macrophages. Cardiomyocyte transplantation increased NK cells and monocytes, while reducing CCR2^−^MHC-II^lo^ macrophages. Consequently, it led to increased mRNA levels of genes involved in extracellular remodelling, poor graft survival, and no functional improvement. Using machine learning-based feature selection, *Mfge8* and *Ccl7* were identified as the primary targets underlying these effects in the heart.

**Conclusions:**

Our results demonstrate that the improved functional outcome following cardiomyocyte transplantation is dependent on a specific CCR2 macrophage response. This work highlights the need to study the role of the immune response for cardiomyocyte cell therapy for successful clinical translation.

**Supplementary Information:**

The online version contains supplementary material available at 10.1186/s13073-023-01213-3.

## Background

Administration of bone marrow-derived stem cells and stem cells from other sources to treat acute myocardial infarction (MI) to thousands of patients over many years has failed to regenerate or completely repair the failing heart [1, 2]. The marginal benefit of these cell transplantation studies has been attributed to paracrine mechanisms. Recently, it has been shown that an acute immune response underlies the benefit of adult stem cell therapy characterized by the temporal and regional induction of CCR2^+^ and CX3CR1^+^ macrophages [3]. This implies that most of the preclinical studies with adult stem cells conducted on immune-deficient animals to maximize the survival and engraftment of the transplanted human cells [4] failed to account for this mechanism of action.

Meanwhile, pluripotent stem cell-derived cardiomyocytes have emerged as a better choice of cell type for transplantation, owing to their evidence in support of medium-term remuscularization [5–8]. However, sustained remuscularization and its mechanism of action is still a point of contention due to the observation that functional benefit persists despite the clearance of the grafted cells [9]. Therefore, a major contribution through paracrine mechanisms seems more likely. Work from other groups have shown that CCR2^−^ and CCR2^+^ macrophages have distinct and crucial roles on cardiac function and healing [10–12]. Studies at the single-cell level have also shown the presence of similar cardiac macrophages [10] and acute inflammatory response after myocardial infarction in humans [13]. In our previous work [14], we showed that syngeneic cardiomyocyte transplantation in wildtype mice altered the cardiac immune response and improved pump function. More specifically, it led to increased cardiac CCR2^+^ macrophage and decreased T_reg_ cell numbers. Interestingly, when combined with short-term pharmacological CCR2 inhibition, the functional benefit was diminished. However, other studies with standalone long-term CCR2 depletion showed improved cardiac function [11, 15], showing that the dynamics of macrophage response has a major impact on cardiac function.

Based on our previous work and current knowledge, we hypothesize that the innate immune response, more specifically macrophages, play an important role in the functional improvement observed following cardiomyocyte transplantation after acute MI. The altered immune response similar to adult stem cells could be a major mechanism of action of transplanted cardiomyocytes. Moreover, most of the preclinical studies for assessing cardiomyocyte therapy require and rely on immunocompromised animal models that have altered macrophage dynamics. Therefore, understanding how these differences could potentially influence the functional benefit of cardiomyocyte transplantation would be critical for successful translation of novel pluripotent stem cell-derived and hypoimmune cardiomyocyte therapies in the clinic.

We investigated wildtype C57BL/6 J mice and compared with non-leaky T cell- and B cell-deficient Rag2^del^ mice. Rag2^del^ mice represent a robust model to study the resident CCR2^−^ and monocyte-derived CCR2^+^ macrophage dynamics with an intact myeloid, dendritic, and natural killer cell compartment. We utilized the highly reproducible permanent ligation model to induce myocardial infarction and reflect the clinical conditions that would require cardiomyocyte cell therapy to replace the damaged tissue. Neonatal cardiomyocytes were used over pluripotent stem cell-derived cardiomyocytes to obviate the uncertainties and variabilities with differentiation protocols and cell maturity. The cells were transplanted 3 days following MI during the acute phase, which, in our view, provides the optimal therapeutic window to influence scar formation and further remodelling processes, while avoiding the early debris-clearing phase.

## Methods

### Mice

Eight to 12-week-old male and female C57BL/6J mice (Charles River, Wilmington, MA, USA) and B6.Rag2^del^ mice (Jackson laboratory, Bar Harbor, ME, USA) were used for this study. Prior to use, mice were bred in the animal facility of the Rostock University Medical Centre and maintained in specified pathogen-free conditions with food and water ad libitum. Following surgical procedures, the mice were allowed to recover in individual cages.

### Neonatal cardiomyocyte isolation

Ventricles were harvested from neonatal transgenic B6.eGFP mice, the tissue was manually minced, and single-cell suspensions were prepared using the Pierce primary cardiomyocyte isolation kit (Thermo Fisher Scientific, Waltham, MA, USA), according to the manufacturers protocol.

### Surgical procedure

**Fig. 1:**
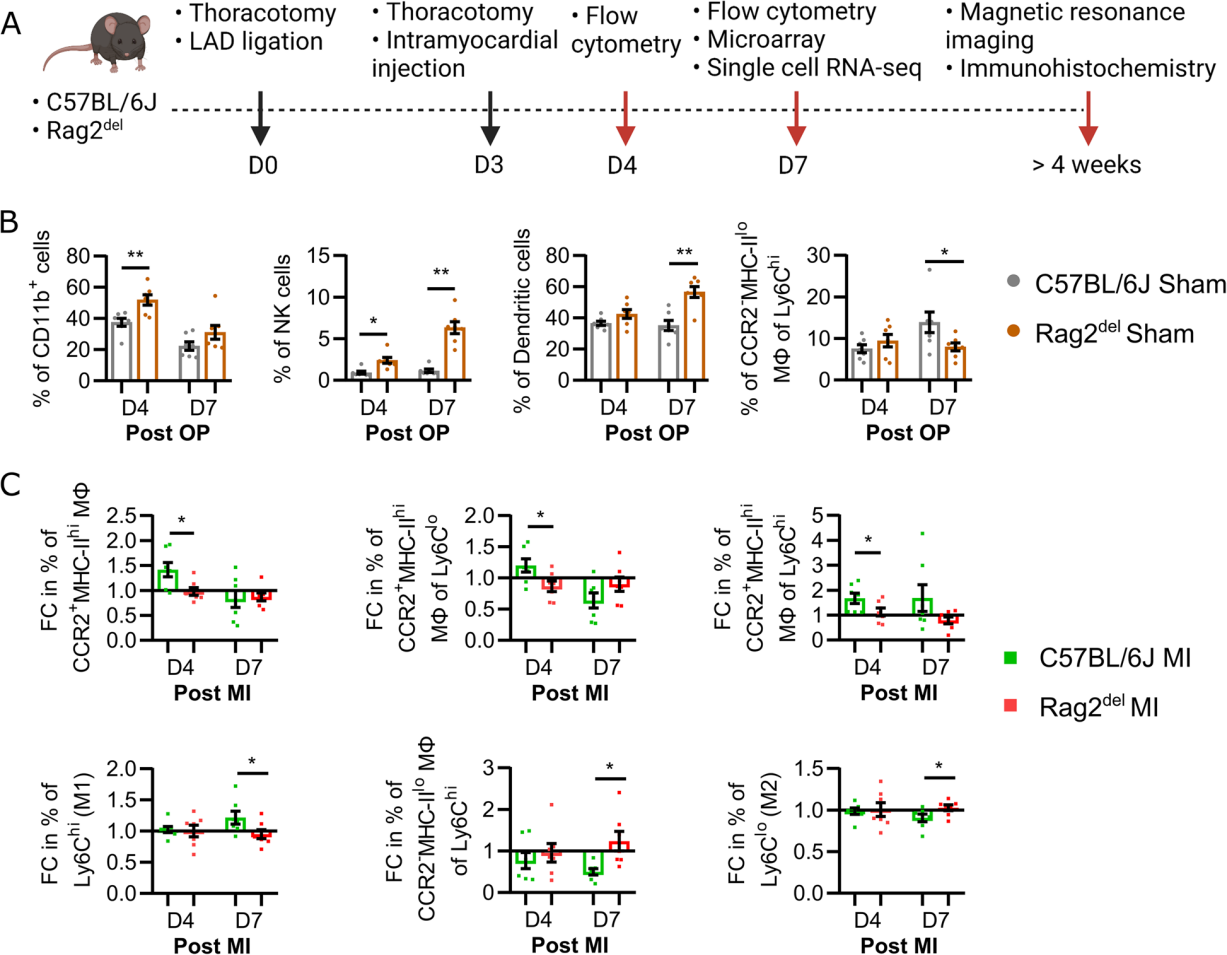
Cardiac innate immune response at steady state and after MI is different between C57BL/6 J and Rag2^del^ mice. **A** Schematic of experimental setup with 8 to 12-week-old male and female C57BL/6 J and Rag2^del^ mice. The sham group underwent two thoracotomies and an intramyocardial injection of Matrigel™. The MI group underwent two thoracotomies, permanent LAD ligation, and intramyocardial injection of Matrigel™. The MI-CM group underwent two thoracotomies, permanent LAD ligation, and intramyocardial injection of 1 million neonatal GFP cardiomyocytes suspended in Matrigel™. Flow cytometric analysis of the cardiac immune response at D4 and D7 after the initial thoracotomy (post OP) or LAD ligation (post MI) was carried out. **B** Differences in the percentage of cells at steady state between the sham groups. **C** Fold change (FC) difference of the percentages of various immune cells in the MI groups compared to the sham groups of respective C57BL/6 J and Rag2.^del^ mice. In both **B** and **C**, values are represented as mean ± SEM. *N* = 7 in all groups and time points. Significance was calculated using the Mann–Whitney test. *p ≤ 0.05

Mice were anesthetized with intraperitoneal administration of pentobarbital (50 mg/kg body weight) and subcutaneous administration of fentanyl (2 μg/kg body weight). The experimental setup is shown in Fig. 1A. Briefly, after intubation and thoracotomy, permanent myocardial infarction was induced through ligation of the left anterior descending coronary artery (LAD). Bleaching of the ventricle was controlled visually to confirm infarction. Three days later, the thorax was reopened, and either 1 × 10^6^ neonatal GFP cardiomyocytes suspended in 15 μl Matrigel™ (MI-CM) or only Matrigel™ (Sham and MI) were injected intramyocardially in four separate injections equidistant along the peri-infarct border zone of the left ventricle. Inhibition of cardiac monocyte infiltration in the MI-CM + I group was carried out by oral administration of a CCR2 inhibitor (RS504393; Sigma-Aldrich, Saint Louis, MO, USA) twice daily (2 mg/kg bodyweight) for 5 days (day 3 to day 7 post MI) accompanied by cell transplantation. The animals in the sham group underwent thoracotomy, placement of a suture in the heart without ligation and reopening of the thorax 3 days later. After completion of the experiments, the mice were euthanized by cervical dislocation and the organs were harvested for further analysis.

### Flow cytometry

Cardiac single immune cell suspensions from entire ventricles were prepared as previously described [14, 16]. Briefly, the ventricles were dissected, enzymatically digested, filtered, treated with RBC lysis buffer, resuspended in MACS® buffer (PBS, 2 mM EDTA, 0.5% BSA), stained for various markers with antibodies listed in Table 1 and analysed on BD FACS LSR II® running BD FACS Diva 8 software (Becton Dickinson, Franklin Lakes, NJ, USA). The different immune cell subpopulations were assessed using a previously described gating strategy [14] as shown in Additional file 1: Fig. S1A.

**Table 1:** List of antibodies used

Target	Clone	Source
CD45	30-F11	Biolegend
CD11b	M1/70	Biolegend
CD11c	N418	Biolegend
NK1.1	PK136	Biolegend
Ly6G	1A8	Biolegend
Ly6C	Hk1.4	Biolegend
CCR2	475301	R and D
MHC II	AF6-120.1	Biolegend
CD3e	145-2C11	Biolegend
CD8a	53–6.7	Biolegend
CD4	RM4-5	Biolegend
FoxP3	MF-14	Biolegend
Anti-CD31	MEC 7.46	Abcam
Anti-CD68	FA-11	Invitrogen
Anti-CCR2	EPR20844-15	Abcam

### Cell sorting and single-cell RNA sequencing

Entire mouse ventricles were isolated and enzymatically digested as described above. Cells were then labelled with CD45 magnetic beads (Miltenyi Biotec, Germany) and positively enriched using the AutoMACS instrument (Miltenyi Biotec, Germany). Viable macrophages/monocytes (CD45^+^CD11b^+^CD11c^−^DAPI^−^Lactadherin^lo^), dendritic cells (CD45^+^CD11b^+^CD11c^+^ DAPI^−^Lactadherin^lo^), and NK cells (CD45^+^CD11b^−^CD11c^+^NK1.1^+^ DAPI^−^Lactadherin^lo^) were then sorted on the BD FACSAria™ IIIu (Becton Dickinson, Franklin Lakes, NJ, USA) roughly in a 1:1:1 ratio into DMEM containing 10% FCS before processing for 10 × Genomics single-cell RNA sequencing (scRNA-Seq).

Single-cell library preparation, sequencing, and analysis was performed as previously described [17]. Briefly, cells were resuspended in PBS with 0.04% BSA and diluted to a concentration of 1000 cells/μl. Cell viability amounted > 91% as assessed using the Cellometer Auto 2000 Cell Viability Counter. Single cells were then captured in droplet emulsions using the GemCode Single-Cell Instrument (10 × Genomics) with a target output of 2500 cells. Libraries for scRNA-Seq were constructed according to the 10 × Genomics protocol using the GemCode Single-Cell 3′ Gel Bead and Library V3 Kit. Quality of amplified cDNA and final libraries were evaluated on the 2100 Bioanalyzer instrument (Agilent) using a High Sensitivity NGS Analysis Kit (Advanced Analytical). Subsequent sequencing was conducted on the HighSeq4000 Sequencing System using the HiSeq SBS and HiSeq PE Cluster Kit V4 (all Illumina, San Die-go, CA. USA). Pre-processing of raw data was conducted by using the CellRanger Software (v.6.0.0) provided by 10 × Genomics. The raw sequencing data is accessible at the EMBL-EBI ArrayExpress website under the accession number E-MTAB-13147 (https://www.ebi.ac.uk/biostudies/arrayexpress/studies?query=+E-MTAB-13147) [18]. The scRNA-seq fastq data files were aligned with STAR [19] (v.2.5.2b) to the mm10 genome (gencode vm23) index, annotated via GTF file, and grouped by barcodes and UMIs resulting in a feature-barcode matrix. Downstream analysis was performed using Seurat [20] (v.4.3.0). After following the standard pipeline of normalization, finding variable features, scaling, and dimensionality reduction by principal-component analysis (PCA), the datasets were merged in a single Seurat object for an integrative analysis. To correct for potential batch effects, the upstream processing algorithm Harmony [21] (v.0.1) was continued before continuing the analysis work flow. The integrated dataset was used for uniform manifold approximation and projection (UMAP) clustering utilizing the formerly generated Harmony embeddings. Differential expression analysis of single-cell clusters was conducted by using deseq2, roc, lr, and *t* tests of Seurat. Only transcripts with a log fold-change higher than 0.25 and *q*-values smaller than 0.05 have been considered as significantly different. Monocle [22] (v.2.2.4) was used to perform sc-RNAseq trajectory analyses, while previously identified clusters have been retained by using the “Seurat-wrappers” (https://github.com/satijalab/seurat-wrappers) pipeline. The underlying R-Script can be obtained via FairdomHub (https://fairdomhub.org/models/825).

### Magnetic resonance imaging (MRI)

MRI measurements and analysis of cardiac pump function and wall thickness were performed as previously described [14, 23]. Briefly, after anaesthesia induction, the mice were placed in supine position and surface coil was placed on the thorax of the mice. Animals were warmed, and respiration rate, heart rate, and body temperature were monitored during the scans. IntraGate gradient-echo cine sequences (IntraGate Cine-FLASH) in short-axis planes were then acquired with a 7 Tesla BioSpec 70/30 (Bruker, Ettlingen, Germany). Data analysis was subsequently carried out using the freely available Segment software [24]. Feature tracking strain analysis was carried out using the strain module.

### Histological analysis

Four weeks after MI, the hearts were arrested in the diastolic phase by administration of 5% KCl, removed and flash frozen in Tissue-Tek® O.C.T. Compound (Sakura Finetek, Alphen aan den Rijn, Netherlands) with liquid nitrogen. Frozen hearts were cryosectioned into 6 μm axial sections divided into five different levels from the apex to the base. For fibrosis estimation, two contiguous sections representing the middle section of the heart were stained with Fast Green FCF (Sigma-Aldrich) and Sirius Red (Chroma Waldeck GmbH & Co. KG, Münster, Germany) and assessed using computerized planimetry (Axio Vision LE Rel. 4.5 software, Carl Zeiss AG, Oberkochen, Germany). The left ventricle was then split into six segments and relative fibrotic area was quantified with Fiji software.

### Immunohistochemistry

The sections were stained as previously described [14]. Briefly, they were fixed in 4% paraformaldehyde, blocked in DAKO protein block (Agilent Technologies, Santa Clara, CA, USA), and labelled using the antibodies mentioned in Table 1. Following secondary antibody addition and mounting with DAKO mounting medium containing DAPI (Agilent Technologies), fluorescence images were obtained using a Zeiss ELYRA PS.1 LSM 780 confocal imaging system (Carl Zeiss AG). The images were then analysed using Fiji software [25].

### RNA isolation and microarray analysis

RNA isolation of cardiomyocytes was carried out using the NucleoSpin® RNA isolation kit (Macherey–Nagel, Düren, Germany) according to the manufacturer instructions. RNA integrity was evaluated using the Agilent Bioanalyzer 2100 with the RNA Pico chip kit (Agilent Technologies). For microarray analysis, 200 ng of isolated RNA samples were applied, and hybridization was performed as described previously [26]. Hybridization was carried out on Affymetrix Clariom™ D Arrays according to the instructions of the manufacturer (Thermo Fisher Scientific). Data analysis was conducted with the provided Transcriptome Analysis Console Software from Thermo Fischer Scientific (Version 4.0.1). The raw microarray data is accessible at the EMBL-EBI ArrayExpress website under the accession number E-MTAB-13152 (https://www.ebi.ac.uk/biostudies/arrayexpress/studies?query=E-MTAB-13152) [27]. The analysis included quality control, data normalization, and statistical testing for differential expression (Limma). Transcripts are considered as significantly differentially expressed (DE) with a fold change (FC) higher than 2 or lower than − 2, false discovery rate (FDR) < 0.5, and *p*-value < 0.05. Gene expression networks were visualized using the Cytoscape application ClueGo [28]. Heatmaps were generated using heatmapper [29].

### Machine learning

Identifying key features and classification of the murine data was obtained by employing supervised and unsupervised machine learning (ML) algorithms [30]. We pre-processed the microarray data, while removing features with low variance for dimension reduction following best practices recommendations. We compared and benchmarked the following supervised algorithms on our specific data: AdaBoost, support vector machines, and random forest (RF) [31]. Small datasets are often prone to overfitting, which is why we employed classifiers that are suitable for training on small data sets for a comparison of features given little training and have chosen the most appropriate algorithm according to accuracy and robustness towards overfitting [32]. Supervised ML models have been tenfold cross-validated, and receiver-operating characteristics (ROC) curves have been generated for binary classifications. We employed PCA, UMAP and, t-distributed stochastic neighbour embedding (t-SNE) for unsupervised ML classification and nonlinear dimensionality reduction [33].

### Statistics

All data are presented as mean values ± standard error of mean (SEM). Mann–Whitney test was used for non-parametric analysis of flow cytometric data. Statistical significance was calculated using a one-way ANOVA, followed by Bonferroni or Dunnett’s post hoc tests for multiple comparisons. For comparison of two groups, unpaired two-tailed *t* test and Mann–Whitney test was applied. Microarray data were tested using Limma and ANOVA with Bonferroni post hoc correction for expression fold change comparison of ML transcripts. No randomization was used in this study. Probability levels considered as statistically significant were *p ≤ 0.05, **p ≤ 0.01, and ***p ≤ 0.001. Calculations and graph analysis were performed using the GraphPad Prism software (GraphPad Prism, Inc., San Diego, CA, USA).

## Results

### *Cardiac innate immune response at steady state and after MI is different between C57BL/6 J and Rag2*^*del*^* mice*

Before evaluating the influence of the innate immune cells on cardiomyocyte transplantation, we studied the differences in the baseline steady state cardiac innate immune response of C57BL/6 J and Rag2^del^ mice. Both these mice lines were subjected to thoracotomy and placement of a suture over the LAD without ligation followed by intramyocardial Matrigel™ injection (Sham) 3 days after the initial thoracotomy. The immune cells isolated from the heart, 4 and 7 days following the initial thoracotomy (post OP), were analysed using flow cytometry (Fig. 1B).

Four days post OP, Rag2^del^ mice had a significantly higher number of CD11b^+^ myeloid cells and natural killer (NK) cells, when compared to C57BL/6 J mice. Seven days post OP, they had a higher number of dendritic cells and NK cells but a lower number of CCR2^−^MHC-II^lo^ macrophages of the proinflammatory Ly6C^hi^ pool.

In order to understand the differences in the immune response after myocardial infarction (MI), we assessed the fold change (FC) difference of the various immune cells in the MI groups compared to the Sham groups of respective C57BL/6 J and Rag2^del^ mice (Fig. 1C). Four days after MI, Rag2^del^ mice showed a decrease in the number of CCR2^+^MHC-II^hi^ macrophages as well as their anti-inflammatory Ly6C^lo^ and proinflammatory Ly6C^hi^ subset response. Seven days after MI, even though there was an overall decreased pro-inflammatory Ly6C^hi^ and increased anti-inflammatory Ly6C^lo^ macrophage response, the CCR2^−^MHC-II^lo^ macrophages of the proinflammatory Ly6C^hi^ pool were increased in Rag2^del^ mice when compared to C57BL/6 J mice.

### Single-cell RNA sequencing analyses reveals strain-dependent differences in the cardiac CCR2 macrophage gene expression profile after MI

**Fig. 2:**
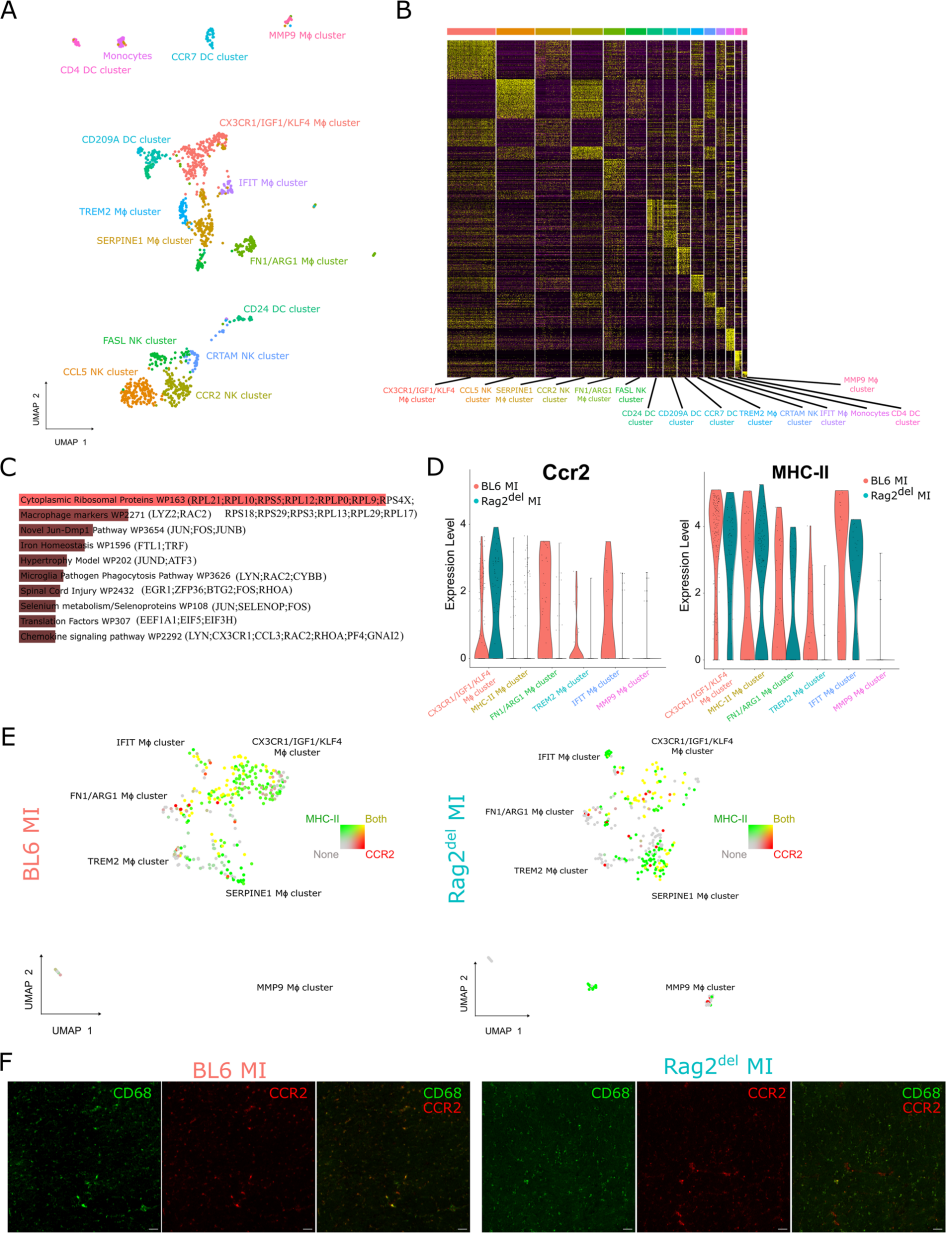
Single-cell RNA sequencing analyses reveals strain-dependent differences in innate immune cells after MI. **A** Single-cell transcriptomic analysis was performed on these sorted cells as mentioned in Figure S1.B using the 10 × Genomics platform. UMAP dimensionality reduction analysis identified 15 major cell clusters as indicated. **B** The top 30 cluster-defining genes were used to generate a similarity score to the different immune cell populations found in the ImmGen repository. **C** Pathway enrichment analysis of the differentially expressed genes between C57BL/6 J and Rag2^del^ using WikiPathways along with **D** violin plots of *CCR2* and *MHC-II* expression levels in the various macrophage clusters. **E** UMAP visualization of the distribution of *CCR2* and *MHC-II* expressing cells in the macrophage clusters. **F** Visualization of CD68 + (green) and CCR2 + (red) cells in the heart 4 weeks after MI. Scale bar represents 20 μm

We then proceeded to understand the transcriptomic differences of the innate immune cells using single-cell RNA sequencing. For this, we sorted CD45^+^CD11b^+^CD11c^−^ macrophages/monocytes, CD45^+^CD11b^+^CD11c^+^ dendritic cells, and CD45^+^CD11b^−^CD11c^+^NK1.1^+^ NK cells (Additional file 1: Fig. S1B) and pooled them together. Dead and apoptotic cells were excluded using DAPI and Lactadherin respectively. scRNA-seq was then performed on these cells using the 10 × Genomics platform. UMAP dimensionality reduction analysis identified 15 different macrophage, dendritic, and NK clusters (Fig. 2A). To validate the cluster identity, we used the top 30 cluster-defining genes to generate a similarity score to the different immune cell populations found in the ImmGen [34] repository (Fig. 2B). We confirmed six macrophage clusters (CX3CR1/IGF1/KLF4, FN1/ARG1, IFIT, SERPINE1, TREM2, and MMP9), one monocyte cluster, four dendritic clusters (CCR7, CD4, CD24, and CD209A), and four NK clusters (CCL5, CCR2, CRTAM, and FASL).

We then analysed the overall differential gene expression between innate immune cells from C57BL/6 J and Rag2^del^ mice. Additional file 1: Fig. S2A reveals the top differentially expressed (DE) genes in the different cell clusters between the two mice models. Enrichment analysis of the DE genes revealed significant differences in macrophage markers (*Lyz2*, *Rac2*), chemokine signalling pathway (*Lyn*, *Cx3cr1*, *Ccl3*, *Rac2*, *Rhoa*, *PF4*, *Gnai2*), and iron homeostasis (*Ftl1*, *Trf*) (Fig. 2C). This finding pointed us to look more closely at the differences between the various macrophage clusters (Additional file 1: Fig. S2B). The CX3CR1/IGF1/KLF4 cluster primarily expressed varied macrophage marker genes involved in cell detection and activation (*CD86*, *CD74*, *CD163*, *CD83*, *Lyz2*, *CD14*) and complement activation (*C1qb*, *C4b*, *C1qa*, *C1qc*). The FN1/ARG1 cluster predominantly expressed genes involved in inflammatory response (*CD80*, *Fn1*, *Thbs1*) and chemokine signalling pathway (*Ccr1*, *Ccl9*, *Ncf1*, *Ccl6*, *Ccl17*). The IFIT macrophage cluster expressed mainly genes of type II interferon signalling (*Cxcl10*, *Isg15*, *Ifit2*) and chemokine signalling (*Cxcl10*, *Ccl12*), whereas genes involved in Keap1-Nrf2 pathway (*Gclm*, *Nfe2l2*) and selenium metabolism (*Selenoh*, *Nfe2l2*, *Msrb1*) were expressed by the SERPINE1 macrophage cluster. Meanwhile, the TREM2 cluster expressed genes involved in iron homeostasis (*Ftl1*), PPAR signalling pathway (*Slc27a1*, *Fabp5*) and oxidative stress (*Mt1*). The MMP9 cluster mainly expressed the matrix metalloproteinase *Mmp9*.

Considering that cardiomyocyte transplantation altered the cardiac CCR2^+^MHC-II^hi^ response in our previous work [14], we sought to analyse the expression of CCR2 and MHC-II in these clusters. It could be seen from Fig. 2D and 2E that, in Rag2^del^ mice, only the CX3CR1/IGF1/KLF4 cluster expressed high levels of *CCR2*, whereas in C57BL/6 J mice, the FN1/ARG1, TREM2, and IFIT macrophage clusters also expressed *CCR2*. *MHC-II* expression was similar in most of the clusters in both mice strains. The CCR2^+^MHC-II^hi^ macrophages between C57BL/6 J and Rag2^del^ mice also had differential gene expression related to a variety of inflammatory pathways and processes (Additional file 1: Fig. S2C). Even after 4 weeks, varied expression and prevalence of CCR2 and CD68 macrophages could be observed between the mice strains (Fig. 2F).

### *Rag2*^*del*^* mice demonstrate an attenuated CCR2 macrophage and increased monocyte response following cardiomyocyte transplantation*

**Fig. 3:**
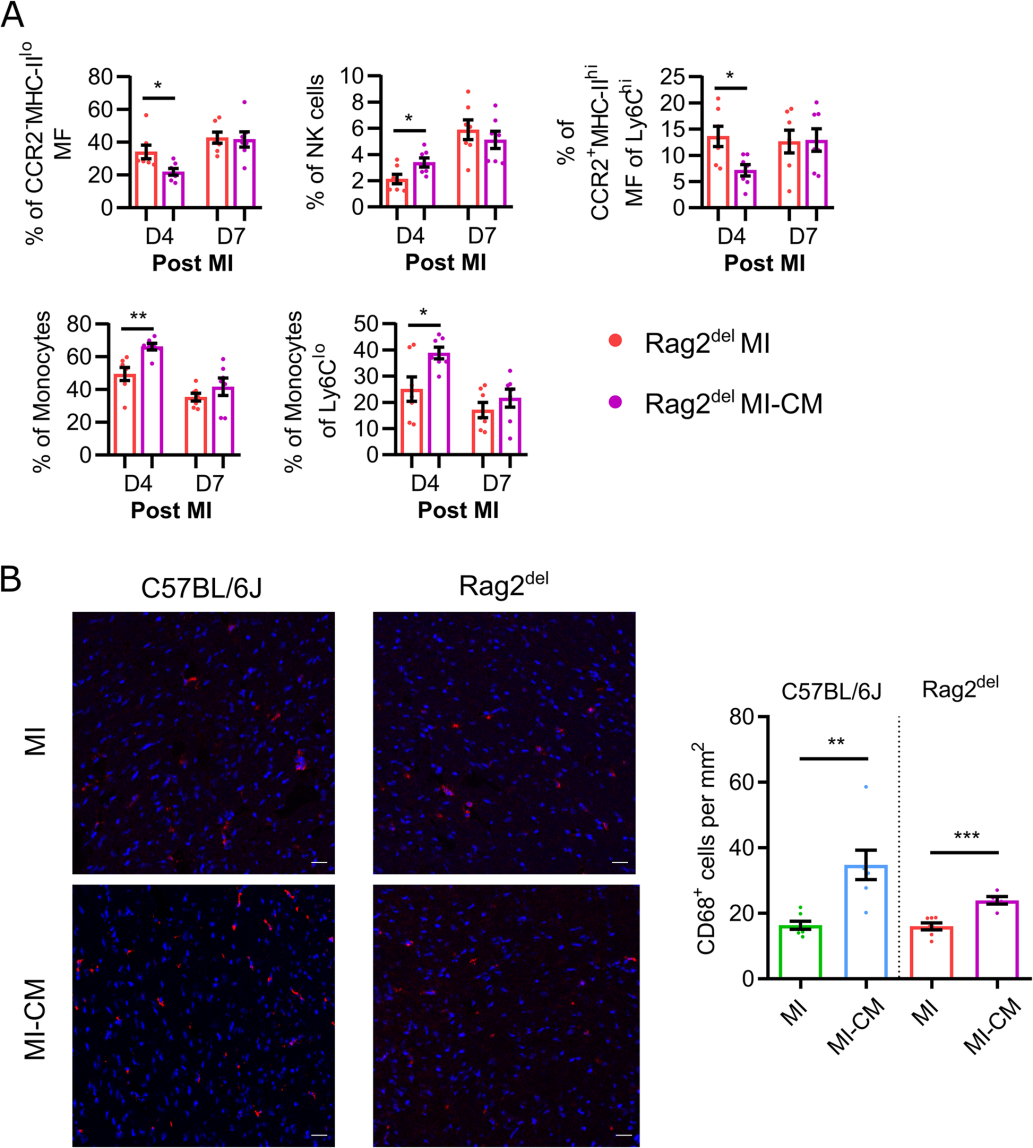
Rag2^del^ mice demonstrate an attenuated CCR2 macrophage and increased monocyte response following cardiomyocyte transplantation. **A** Flow cytometric analysis of the cardiac immune response at D4 and D7 post MI. Values are represented as mean ± SEM. *N* = 7 in all groups and time points. Significance was calculated using the Mann–Whitney test. *p ≤ 0.05, **p ≤ 0.01. **B** Assessment of CD68^+^ cells in the remote area of the heart 4 weeks after MI. CD68^+^ cells are stained red and the nuclei are stained blue using DAPI. Scale bar represents 20 μm. ‘MI’ refers to the infarct group, and ‘MI-CM’ refers to the cell transplanted group. Values are represented as mean ± SEM. *N* = 7,7,7,5. Significance between the respective MI and MI-CM groups were calculated using an unpaired two-tailed *t* test. **p ≤ 0.01, ***p ≤ 0.001

Given the inherent differences observed above in the innate immune response, we set out to investigate the changes in the cardiac immune response following cardiomyocyte transplantation in Rag2^del^ mice using flow cytometry. Our previous work showed that it reduced the number of CCR2^+^MHC-II^hi^ macrophages in C57BL/6 J mice [14]. Four days after MI and 1 day following cell therapy, we observed a significant decrease in CCR2^−^MHC-II^lo^ as well as CCR2^+^MHC-II^hi^ macrophages of Ly6C^hi^ pool with a complementary increase in NK cells, monocytes, and their relative contribution to the Ly6C^lo^ subsets in the cardiomyocyte treated group compared to the MI control (Fig. 3A).

In order to understand whether there were changes in the macrophage numbers over a longer period of time, we quantified the number of CD68^+^ macrophages in the remote (Uninfarcted) area of the heart, 4 weeks after MI and cardiomyocyte transplantation (Fig. 3B). We found almost 2.5 times more CD68^+^ cells in the cell treated C57BL/6 J mice, while the cell treated Rag2^del^ mice had 1.5 times more, when compared to their MI controls.

### Altered innate immune response following cardiomyocyte transplantation fails to improve cardiac function

We then went forward to investigate and compare the graft survival and functional outcome following the altered innate immune response observed between wildtype C57BL/6 J and Rag2^del^ mice. Accordingly, cardiac pump function and morphology was assessed four weeks after thoracotomy/MI using the highly sensitive magnetic resonance imaging technology, thus requiring fewer animals per group. Meanwhile, graft survival, fibrosis and capillary density was assessed using immunohistochemistry.

**Fig. 4:**
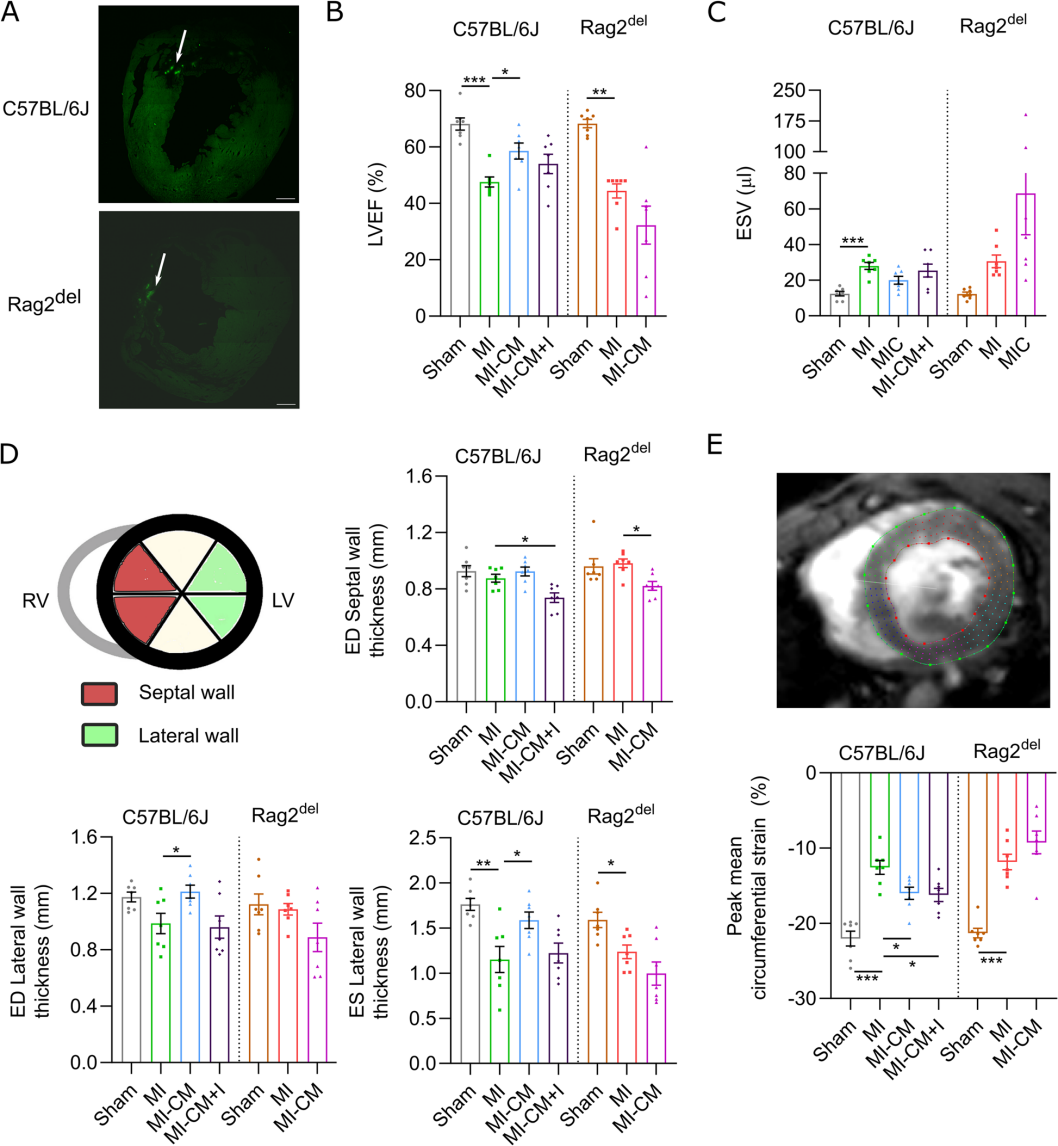
Altered innate immune response following cardiomyocyte transplantation fails to improve cardiac function. **A** Representative tile scan of the heart 4 weeks after cell transplantation with an arrow pointing towards GFP signals observed at the injection site. Scale bar represents 400 μm. **B** Assessment of left ventricular ejection fraction (LVEF, %) and **C** end-systolic volume (ESV, μl) 4 weeks after MI/thoracotomy using MRI. **D** Changes in cardiac remodelling was monitored by assessing the left ventricular wall thickness from MRI images. **E** Strain analysis of the hearts was performed with the MRI Cine images using the feature-tracking strain analysis module and the peak mean circumferential strain was calculated. ‘MI’ refers to the infarct group, ‘MI-CM’ refers to the cell transplanted group, and ‘MI-CM + I’ refers to the cell transplantation combined with CCR2 inhibition. Values are represented as mean ± SEM. *N* = 7 in all groups. Significance was calculated separately for C57BL/6 J and Rag2^del^ mice using one-way ANOVA with Dunnett’s post hoc test for multiple comparisons. *p ≤ 0.05, **p ≤ 0.01, ***p ≤ 0.001

In order to study graft survival, we evaluated the presence of transplanted GFP cardiomyocytes in the hearts, 4 weeks after MI. GFP signals were observed from the injection site (Fig. 4A) in 3 out of 7 C57BL/6 J hearts and only in 1 out of 7 Rag2^del^ hearts.

On further analysis of cardiac pump function, we observed a slight decline in left ventricular ejection fraction (LVEF) (Fig. 4B) and end-systolic volume (ESV) (Fig. 4C) in Rag2^del^ mice, whereas significant improvement in LVEF was observed in C57BL/6 J mice following cardiomyocyte transplantation compared to their infarct controls. Incidentally, when combined with pharmacological CCR2-inhibtion, there was no significant improvement in LVEF in C57BL/6 J mice following cardiomyocyte transplantation.

In order to understand ventricle remodelling and subsequent changes in the infarct wall, we performed a comprehensive quantitative regional wall analysis of the left ventricle from the MRI images (Fig. 4D). Concurring with the deteriorated LVEF and ESV values, Rag2^del^ mice showed thinner end diastolic (ED) septal and end systolic (ES) lateral walls following cardiomyocyte transplantation. Meanwhile, the improved pump function in cell treated C57BL/6 J mice agreed with thicker ES and ED lateral walls. However, when combined with CCR2-inhibition, it led to thinner septal and lateral walls similar to Rag2^del^ mice.

To further understand the mechanistics of the observed functional differences following cardiomyocyte transplantation, we performed circumferential strain analysis of the left ventricle from short-axis MRI measurements to deduce the changes in cardiac muscle contractility (Fig. 4E). It could be clearly seen that there was a significant improvement in cardiac contractility as a measure of peak mean circumferential strain only in C57BL/6 J mice and a slight decline in Rag2^del^ mice. Interestingly, combined with CCR2-inhibition in C57BL/6 J mice, cardiomyocyte transplantation still led to significant improvement in cardiac muscle contractility.

In order to understand the effect on cardiac fibrosis, the relative fibrotic area in the heart was assessed using Sirius Red staining (Additional file 1: Fig. S3A). We observed no significant difference following cardiomyocyte transplantation in both C57BL/6 J and Rag2^del^ hearts. Similarly, no improvement in capillary density was seen as assessed by CD31 staining (Additional file 1: Fig. S3B) in the remote (Uninfarcted) area in both C57BL/6 J and Rag2^del^ mice.

### Transcriptomic profiling reveals changes in inflammatory and extracellular remodelling pathways

**Fig. 5:**
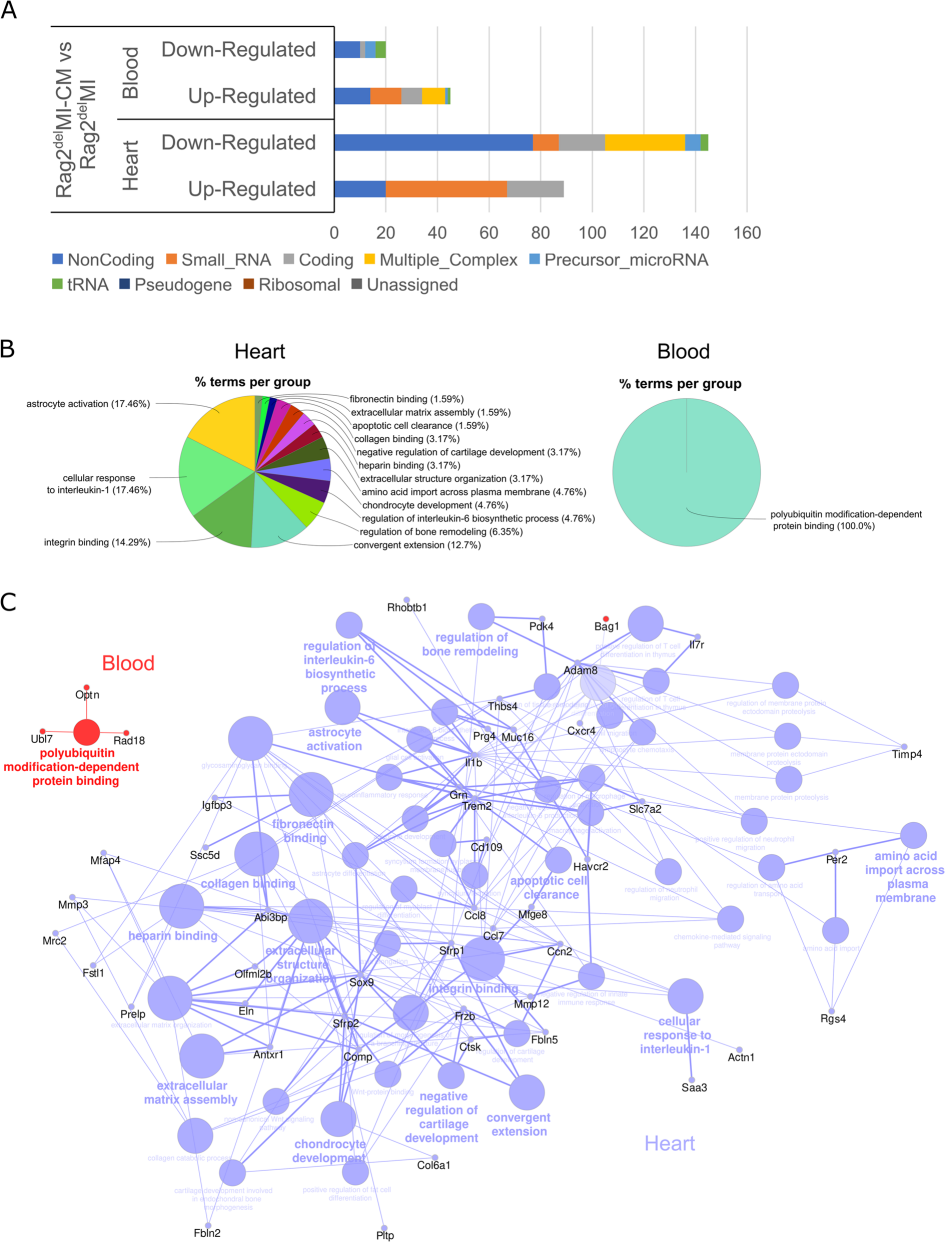
Transcriptomic profiling reveals changes in inflammatory and extracellular remodelling pathways. Rag2^del^ mice were subjected to MI (denoted as MI) with or without cardiomyocyte transplantation (MI-CM), and 7 days after MI, the RNA from the heart and blood was isolated and analysed via a Clariom™ D microarray. **A** Analysis of the relative contribution of the various RNA categories of the upregulated and downregulated genes revealed a substantial contribution from non-coding RNAs (ncRNAs). Transcripts are considered differentially expressed (DE), if they show a twofold change and have a *p*-value < 0.05. **B** GO enrichment analysis expressed as % terms per group was performed on DE targets in the heart and blood. **C** Network analysis using the GO terms of these targets. In the network, the transcripts from the heart and the corresponding GO terms are represented in blue circles with the transcripts from the blood and their corresponding GO terms being represented in red circles. *N* = 3 in both groups

We then proceeded to analyse the gene expression pathways as a result of the altered innate immune response and the deteriorated functional outcome in Rag2^del^ mice and examine whether there is a systemic component to the observed changes. The transcriptional profile of the heart and blood was assessed, 7 days after MI in both the untreated and cell treated groups using Clariom™ D microarrays. Analysis of the relative contribution of the various RNA categories revealed a substantial contribution from non-coding RNAs (ncRNAs) that are downregulated in the heart (Fig. 5A). The heatmaps reveal the DE transcripts (twofold or greater with *p* < 0.05) between the cell treated (MI-CM) and MI control (Additional file 1: Fig. S4).

GO enrichment analysis of the DE transcripts (Fig. 5B) showed genes enriched in cellular response to interleukin-1 and integrin binding along with a multitude of pathways involved in extracellular remodelling in the heart. In contrast, only polyubiquitin modification-dependent protein binding was enriched in the blood. Network analysis of these enriched genes (Fig. 5C) shows the complex interplay between the various biological processes and the bridging roles played by *Abi3bp*, *Adam8*, *Ccl7*, *Ccl8*, *Comp*, *Il1b*, *Mfge8*, *Sfrp2*, and *Trem2*. An eminent mediator of this extracellular remodelling can be attributed to the increased *Thbs4* expression in the heart, which has been shown to promote fibrosis and extracellular matrix disassembly following ischemia [35]. The increased expression of *MMP12* and *Timp4* but lowered *MMP3* expression in the cell treated group reveals the interplay of various matrix metalloproteinases in increased degradation of elastin and thus reduction of elasticity of the cardiac muscle. The inflammation is modulated by reduction in *Il1b*, *Ccl7*, and *Ccl8* but increase in *CD68*, *CD109*, *Il7r*, and *Cxcr4* levels. A list of the GO terms is provided in Additional file 1: Fig. S5. The amounts of DE transcripts in the blood are small and the immune response is affected mainly through increased *Optn* and *Ogdh* levels in the MI-CM group.

### Machine learning identifies the most important targets of cardiomyocyte transplantation and their functional outcome

In order to further identify the targets of cardiomyocyte transplantation and their functional outcome, we analysed the DE genes of untreated and cell treated groups in both C57BL/6 J and Rag2^del^ mice. A machine learning (ML) based feature selection approach was applied to independently rank the importance of the identified significant DE transcripts [36]. The resulting ML model was cross-validated. Consequently, we identified a high correlation between the respective gene expression and cell transplantation, thus suggesting these transcripts as important factors for the mechanism of action of transplanted cardiomyocytes. Transcription factors are marked with a ‘*’ next to their name.

**Fig. 6:**
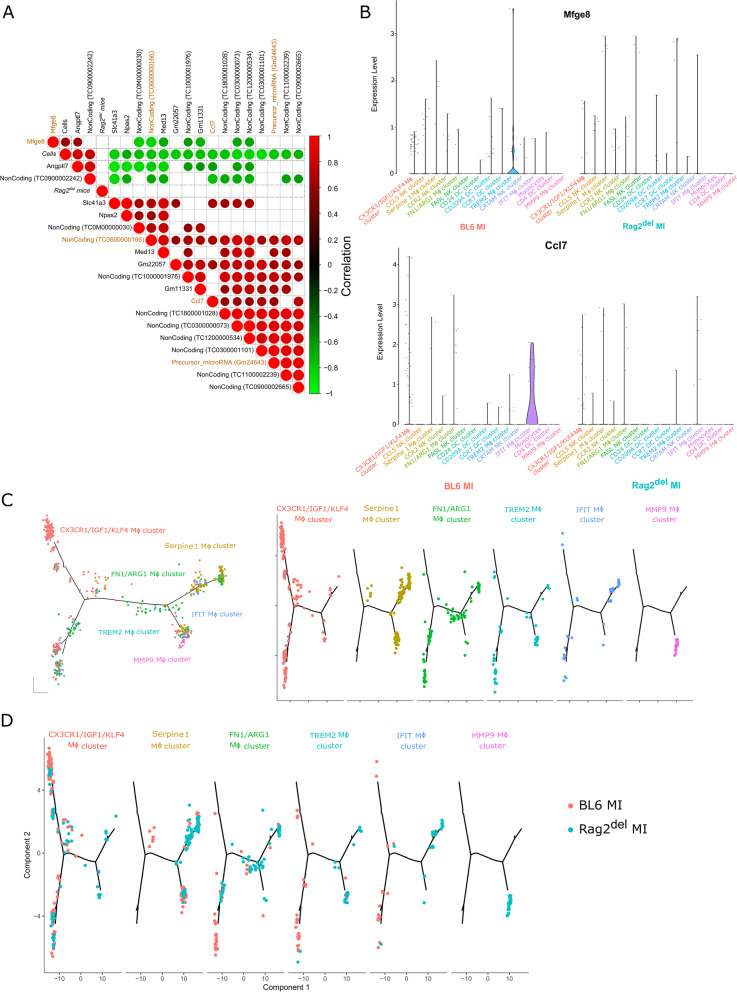
Machine learning and high dimensional single-cell mapping and trajectory analysis identifies the most important targets of cardiomyocyte transplantation and hints at differences in macrophage development. **A** The differentially expressed transcripts between cell treated mice and untreated mice were compared using the random forest (RF) algorithm to identify the features that are important for cell transplantation (‘Cells ‘) in the heart. The most effective combination of targets is marked in gold. Positive correlation is marked with red circles and negative correlation is marked with green circles (only significant correlations are shown). **B** Violin plots of *Mfge8* and *Ccl7* expression levels in the various single-cell clusters of immune cells after MI in both C57BL/6 J and Rag2^del^ hearts. **C** DE genes between the macrophage clusters from Fig. 2B were used to generate hypothetical developmental relationships using Monocle algorithm. Each line represents individual branches in Monocle with the macrophage subsets occupying distinct branches based on their development. **D** The varied developmental kinetics of the various macrophage subsets between C57BL/6 J and Rag2^del^ mice is shown

In the heart (Fig. 6A), we identified Npas2*, Med13*, which are transcription factors; *Mfge8*, a soluble glycoprotein; *Slc41a3*, a mitochondrial Mg^2+^ transporter along with *Ccl7* (otherwise known as Mcp-3), a chemokine for macrophage infiltration; and other ncRNAs as important targets of cardiomyocyte transplantation. Three transcripts (*Mfge8*, *Angptl7*, and ncRNA: *TC0900002242*) positively correlate to cardiomyocyte transplantation with the other 17 targets being negatively correlated. On further scrutiny, the combination of the transcripts *Mfge8*, *Ccl7*, precursor miRNA: *Gm24643*, and ncRNA: *TC0600000166* were able to predict the functional outcome of cardiomyocyte transplantation with 92.4% accuracy (AUC 91.6%) (Additional file 1: Fig. S6A). On checking the sequence similarity and relevance to humans (Additional file 1: Fig. S6C), Gm24643 has only a low predicted conservation rate and no miRNA-like hairpin regions but is close to the predicted binding sites of Stat1*, Gata1*, Foxi1*, and Foxd3*. Meanwhile, the ncRNA (*TC0600000166*) refers to the *Kcnd2* transcript in humans, including multiple predicted mir-like hairpin structures.

### High dimensional single-cell mapping and trajectory analysis hints at differences in macrophage development

In order to understand the contribution of the different immune cells to their expression, we then analysed the expression of *Mfge8* and *Ccl7* in the different cardiac immune single-cell clusters after MI (Fig. 6B). *Mfge8* expression was observed only in the TREM2 macrophage cluster, while only the IFIT macrophage cluster expressed *Ccl7*. Also, both their expression was observed only in C57BL/6 J mice. We surmised that this might be due to the different developmental paths taken by the macrophage subsets and proceeded to analyse their hypothetical developmental relationship using Monocle. We re-clustered the macrophage subsets shown in Fig. 2 to provide single-cell based gene expression change trajectories (Fig. 6C). It revealed that the macrophage populations have distinct patterns on the branches. Moreover, these patterns especially of both the TREM2 and IFIT macrophages were different between the C57BL/6 J and Rag2^del^ mice (Fig. 6D).

## Discussion

The failure of clinical studies involving adult stem cells to regenerate damaged heart tissue could potentially be explained due to a lack of mechanistic understanding of the underlying biological effect as well as discounting the importance of the acute immune response in mediating their benefit [3]. On a similar note, most of the current knowledge on the efficacy of cardiomyocyte transplantation and their mechanism of action does not take the importance of the immune system into account. This work shows that the innate immune response has a major role in mediating the positive improvement in ventricular remodelling, cardiac contractility, and pump function following cardiomyocyte transplantation. Significant functional improvement was observed in C57BL/6 J mice despite the relatively poor engraftment of the transplanted cells. The absence of any significant improvement in Rag2^del^ mice can be partially attributed to the decrease in both CCR2^−^MHC-II^lo^ and anti-inflammatory subset of CCR2^+^MHC-II^hi^ macrophages. While a decrease in CCR2^+^MHC-II^hi^ macrophages was observed 1 day after cell transplantation in C57BL/6 J mice [14], short-term sustained CCR2 inhibition together with cardiomyocyte transplantation resulted in diminished functional benefit, which is marked by retained contractility improvement, but a lack of ventricular wall thickness improvement. This is in agreement with previous standalone immune modulation research attributing decreased CCR2^−^MHC-II^lo^ macrophage and disturbed CCR2^+^MHC-II^hi^ dynamics to adverse cardiac remodelling and pump function [10–12].

These results become particularly important in the context of SCID mice being commonly used in testing cell therapies, in spite of macrophages being dysfunctional. Instead, we utilized Rag2^del^ mice with intact macrophages and NK cells as the immunocompromised model, since our previous work showed cardiomyocyte transplantation to alter the cardiac macrophage response [14]. High-dimensional single-cell mapping and trajectory analysis hinted at differences in macrophage development in Rag2^del^ mice that may underlie the observed differences. Based on the adverse outcomes observed, it can be seen that an increased inflammatory potential, especially NK cells, potentially led to the observance of fewer transplanted cells. Significant differences observed in genes related to antigen processing and presentation (Additional file 1: Fig. S5) indicate that the transplanted cells could be differently recognized and processed, thereby altering the immune response discordantly. This finding is helpful for future studies with human PSC-derived cardiomyocytes as well as hypoimmune cells, where cardiomyocytes with different maturation states, antigenic profiles, and immunogenicity would evoke varied CCR2 macrophage and immune responses. Therefore, our work could be an inflection point for understanding the need to study the role of the immune response for cardiomyocyte therapies.

Interestingly, transplantation of human PSC-derived cardiomyocytes in non-human primate models showed functional improvement [5, 37], despite the varied allogenic and xenogeneic immune responses. Such experiments also use various immunosuppressive regimens. Most of the large animal studies use cyclosporine, whose administration in mice has been shown to negate the beneficial effects of stem cell transplantation [3]. Other immunosuppressive drugs like calcineurin and mTOR inhibitors have different effects on the T cell and B cell response [38–41], while their effect on macrophages are not yet clearly understood. Buprenorphine and carprofen, commonly used as analgesics, also alter the immune system [42–45]. Therefore, it is very likely that the benefits from macrophage response are either minimized or altered in these studies. The use of engineered heart tissue and pro-survival cocktails have addressed the survival of transplanted cells [9]. Moving forward, it would be important to characterize the cardiac immune response in inducible immune deficient, immunosuppressive, and humanized mouse models in order to understand their inherent differences and the observed changes following cell therapy.

Additionally, our ML-based approach identified *Mfge8*, *Ccl7* (commonly known as Mcp-3), precursor miRNA: *Gm24643*, and ncRNA: *TC0600000166* to be the primary targets for the observed effects in the heart following cardiomyocyte transplantation. Moreover, *Mfge8 and Ccl7* expression after MI was observed in TREM2 and IFIT macrophages (both also expressing *CCR2* and *MHC-II*) respectively in C57BL/6 J mice (Fig. 6B). While Ccl7 plays a critical role in CCR2-dependent monocyte recruitment to the heart [46, 47], MFGE8 is involved in cardiac hypertrophy [48], cardiac fibrosis [49], and removal of dead cells [50]. Combined with evidence from single cell and CCR2 inhibition experiments, one could speculate CCR2^+^MHC-II^hi^ macrophages being the prominent effector cells of these targets through which cardiac remodelling and function could be impacted. However, this should be further validated and other cellular sources of *Mfge8* and *Ccl7*, like fibroblasts and cardiomyocytes need to be investigated as well.

There are, however, some limitations to our study. While we used the permanent LAD ligation model to have reproducible and large infarcts, it does not reflect the most representative clinical scenario, where patients undergo revascularization. The survival and integration of the transplanted cells at multiple timepoints were not investigated in detail in this study and further experiments are required to understand the role of the increased NK cell response in influencing cell survival and integration. In order to understand the cell specific effects, further controls are necessary. In addition, other immune deficient models with unique genetic backgrounds need to be investigated to understand the role of immune cell development on the immune response following cardiomyocyte transplantation. The transcriptomic analysis of the influence of cardiomyocyte transplantation on the immune response in this study was done using bulk RNA sequencing, while supplementing it with spatial transcriptomics would reveal locally resolved details of the process. Only with high-throughput spatially resolved single-cell sequencing, which is commercially not yet available, could the direct effects of cardiomyocyte transplantation on the immune cells be established. This study is heavily based on RNA sequencing and flow cytometry and further orthogonal and functional approaches are necessary to validate the identified cells and targets. The ML models used were selected towards their reasonable performance on small datasets and encapsulated the analysis into a more comprehensive experimental scheme and independently validated the results. However, a larger dataset is necessary for a comprehensive ML-based analysis. Also, a more thorough investigation of the various factors identified in our ML-based analysis, using different knockout models, needs to be carried out to understand their relative significance. Finally, it is important to acknowledge the significant differences in the immune response of specific pathogen-free laboratory mice [51–53] that lead to high failure rate in clinical translation. Therefore, models such as the ‘wildlings’ [54] mice that more faithfully reproduce human disease mechanisms by combining wild mouse microbiota with naturally occurring pathogens could be used to increase the translatability of immunological results.

## Conclusions

In summary, this work demonstrates the differences in the innate immune response after myocardial infarction between C57BL/6 J and T cell and B cell deficient Rag2^del^ mice at a single-cell resolution. Furthermore, following cardiomyocyte transplantation, differences in the CCR2 macrophage and NK response dynamics influences graft survival, ventricular remodelling, cardiac contractility, and eventually pump function, with *Mfge8* and *Ccl7* being identified as the primary targets in this scenario. Differences in macrophage development could potentially explain the varied *Mfge8* and *Ccl7* expression in Rag2^del^ mice, thereby asserting the complexity of using genetically immune-deficient mice. Therefore, elaborate studies with clinically established immunosuppression regimens in relevant mouse models in forthcoming research are necessary for successful clinical translation in humans. Hereby, utmost attention should be given to decipher the CCR2 macrophage response and associated paracrine effects following PSC-derived cardiomyocyte as well as hypoimmune cells transplantation.

### Supplementary Information

**Additional file 1: Supplement figures.** **Figure S1.** Representative gating strategy for identifying the cardiac immune cells using flow cytometry and sorting them for single cell RNA sequencing. **Figure S2.** Single-cell RNA sequencing reveals the differentially expressed genes of the various cardiac immune cell clusters between C57BL/6J and Rag2^del^ mice after MI. **Figure S3.** Assessment of cardiac ventricular remodelling. **Figure S4.** Differentially expressed transcripts in the heart and blood. **Figure S5.** GO terms for the DE transcripts in the heart and blood between Rag2^del^MI and Rag2^del^MI-CM groups. **Figure S6.** The most significant transcripts obtained using machine learning feature selection.

## Data Availability

All raw data generated or analysed in this study are available from the corresponding author upon reasonable request, as per FAIR principles. The underlying computational scripts can be obtained from FairdomHub (https://fairdomhub.org/models/825). The raw single cell RNA sequencing data and microarray data generated and analysed in this study are accessible at the EMBL-EBI ArrayExpress website under the accession number E-MTAB-13147 (https://www.ebi.ac.uk/biostudies/arrayexpress/studies?query=+E-MTAB-13147) [18] and E-MTAB-13152 (https://www.ebi.ac.uk/biostudies/arrayexpress/studies?query=E-MTAB-13152)  [27] respectively.

## Abbreviations

RBC: Red blood cells
PCA: Principal-component analysis
PBS: Phosphate buffer saline
EDTA: Ethylenediaminetetraacetic acid
BSA: Bovine serum albumin
MI: Myocardial infarction
MSC: Mesenchymal stem cells
NK: Natural killer
iPSC: Induced pluripotent stem cells
LAD: Left anterior descending coronary artery
MRI: Magnetic resonance imaging
LVEF: Left ventricular ejection fraction
LV: Left ventricle
FC: Fold change
GO: Gene ontology
ML: Machine learning
ROC: Receiver-operating-characteristics
RF: Random forest
UMAP: Uniform manifold approximation and projection
SEM: Standard error of mean
ESV: End-systolic volume
EDV: End diastolic volume
MNC: Mononuclear cells
ncRNA: Non-coding RNA
DE: Differentially expressed

## References

[1] A futile cycle in cell therapy. Nat Biotechnol. 2017;35(4):291. Available from: http://www.ncbi.nlm.nih.gov/pubmed/28398319. 10.1038/nbt.3857 28398319

[2] Bolli R (2020). Cell therapy for acute myocardial infarction: requiescat in pace. Eur Heart J..

[3] Vagnozzi RJ, Maillet M, Sargent MA, Khalil H, Johansen AKZ, Schwanekamp JA (2020). An acute immune response underlies the benefit of cardiac stem cell therapy. Nature.

[4] Lang CI, Wolfien M, Langenbach A, Müller P, Wolkenhauer O, Yavari A (2017). Cardiac cell therapies for the treatment of acute myocardial infarction: a meta-analysis from mouse studies. Cell Physiol Biochem..

[5] Chong JJH, Yang X, Don CW, Minami E, Liu Y-W, Weyers JJ (2014). Human embryonic-stem-cell-derived cardiomyocytes regenerate non-human primate hearts. Nature..

[6] Riegler J, Tiburcy M, Ebert A, Tzatzalos E, Raaz U, Abilez OJ (2015). Human engineered heart muscles engraft and survive long term in a rodent myocardial infarction model. Circ Res..

[7] Weinberger F, Breckwoldt K, Pecha S, Kelly A, Geertz B, Starbatty J, et al. Cardiac repair in guinea pigs with human engineered heart tissue from induced pluripotent stem cells. Sci Transl Med. 2016;8(363). Available from: https://www.science.org/doi/10.1126/scitranslmed.aaf8781. 10.1126/scitranslmed.aaf8781 27807283

[8] Romagnuolo R, Masoudpour H, Porta-Sánchez A, Qiang B, Barry J, Laskary A (2019). Human embryonic stem cell-derived cardiomyocytes regenerate the infarcted pig heart but induce ventricular tachyarrhythmias. Stem Cell Reports..

[9] Zhang J, Bolli R, Garry DJ, Marbán E, Menasché P, Zimmermann W-H (2021). Basic and translational research in cardiac repair and regeneration. J Am Coll Cardiol..

[10] Dick SA, Macklin JA, Nejat S, Momen A, Clemente-Casares X, Althagafi MG (2019). Self-renewing resident cardiac macrophages limit adverse remodeling following myocardial infarction. Nat Immunol..

[11] Lavine KJ, Epelman S, Uchida K, Weber KJ, Nichols CG, Schilling JD, et al. Distinct macrophage lineages contribute to disparate patterns of cardiac recovery and remodeling in the neonatal and adult heart. Proc Natl Acad Sci U S A. 2014 [cited 2014 Oct 30];1–6. Available from: http://www.ncbi.nlm.nih.gov/pubmed/25349429. 10.1073/pnas.1406508111 PMC4234568 25349429

[12] Bajpai G, Bredemeyer A, Li W, Zaitsev K, Koenig AL, Lokshina I (2019). Tissue resident CCR2− and CCR2+ cardiac macrophages differentially orchestrate monocyte recruitment and fate specification following myocardial injury. Circ Res..

[13] Ben-Mordechai T, Palevski D, Glucksam-Galnoy Y, Elron-Gross I, Margalit R, Leor J. Targeting macrophage subsets for infarct repair. J Cardiovasc Pharmacol Ther. 2014 [cited 2014 Sep 1]; Available from: http://www.ncbi.nlm.nih.gov/pubmed/24938456. 10.1177/1074248414534916 24938456

[14] Vasudevan P, Wolfien M, Lemcke H, Lang CI, Skorska A, Gaebel R (2020). Cardiomyocyte transplantation after myocardial infarction alters the immune response in the heart. Cells..

[15] Epelman S, Lavine KJ, Beaudin AE, Sojka DK, Carrero JA, Calderon B (2014). Embryonic and adult-derived resident cardiac macrophages are maintained through distinct mechanisms at steady state and during inflammation. Immunity..

[16] Vasudevan, Gaebel, Doering, Mueller, Lemcke, Stenzel (2019). 18F-FDG PET-Based imaging of myocardial inflammation predicts a functional outcome following transplantation of mESC-derived cardiac induced cells in a mouse model of myocardial infarction. Cells..

[17] Galow A-M, Kussauer S, Wolfien M, Brunner RM, Goldammer T, David R, et al. Quality control in scRNA-Seq can discriminate pacemaker cells: the mtRNA bias. Cell Mol Life Sci. 2021; Available from: http://www.ncbi.nlm.nih.gov/pubmed/34427691. 10.1007/s00018-021-03916-5 PMC8558157 34427691

[18] Wolfien M, Vasudevan P. Single-cell RNA-Seq of entire ventricles for C57BL/6J and B6.Rag2del mice after myocardial infarction. BioStudies, E-MTAB-13147, 2023, https://www.ebi.ac.uk/biostudies/arrayexpress/studies/E-MTAB-13147. Accessed 13 July 2023.

[19] Dobin A, Gingeras TR. Mapping RNA-seq Reads with STAR. Curr Protoc Bioinforma [Internet]. 2015 Sep 3;51(1). Available from: https://onlinelibrary.wiley.com/doi/10.1002/0471250953.bi1114s51. 10.1002/0471250953.bi1114s51 PMC4631051 26334920

[20] Stuart T, Butler A, Hoffman P, Hafemeister C, Papalexi E, Mauck WM (2019). Comprehensive integration of single-cell data. Cell..

[21] Korsunsky I, Millard N, Fan J, Slowikowski K, Zhang F, Wei K (2019). Fast, sensitive and accurate integration of single-cell data with Harmony. Nat Methods..

[22] Qiu X, Hill A, Packer J, Lin D, Ma Y-A, Trapnell C (2017). Single-cell mRNA quantification and differential analysis with Census. Nat Methods..

[23] Lang CI, Vasudevan P, Döring P, Gäbel R, Lemcke H, Lindner T (2021). Expedient assessment of post-infarct remodeling by native cardiac magnetic resonance imaging in mice. Sci Rep..

[24] Heiberg E, Sjögren J, Ugander M, Carlsson M, Engblom H, Arheden H (2010). Design and validation of Segment - freely available software for cardiovascular image analysis. BMC Med Imaging..

[25] Schindelin J, Arganda-Carreras I, Frise E, Kaynig V, Longair M, Pietzsch T (2012). Fiji: an open-source platform for biological-image analysis. Nat Methods..

[26] Koczan D, Fitzner B, Zettl UK, Hecker M (2018). Microarray data of transcriptome shifts in blood cell subsets during S1P receptor modulator therapy. Sci Data..

[27] Wolfien M, Vasudevan P. Microarray data for C57BL/6J and B6.Rag2del mice after cardiomyocyte transplantation. BioStudies, E-MTAB-13152, 2023, https://www.ebi.ac.uk/biostudies/arrayexpress/studies/E-MTAB-13152. Accessed 13 July 2023.

[28] Bindea G, Mlecnik B, Hackl H, Charoentong P, Tosolini M, Kirilovsky A (2009). ClueGO: a Cytoscape plug-in to decipher functionally grouped gene ontology and pathway annotation networks. Bioinformatics..

[29] Babicki S, Arndt D, Marcu A, Liang Y, Grant JR, Maciejewski A (2016). Heatmapper: web-enabled heat mapping for all. Nucleic Acids Res..

[30] Kuhn M. Building predictive models in R using the caret package. J Stat Softw. 2008;28(5). Available from: http://www.jstatsoft.org/v28/i05/.

[31] Forman G, Cohen I. Learning from little: comparison of classifiers given little training. In 2004;161–72. Available from: http://link.springer.com/10.1007/978-3-540-30116-5_17.

[32] Saeb ATM, Al-Naqeb D (2016). The impact of evolutionary driving forces on human complex diseases: a population genetics approach. Scientifica (Cairo).

[33] van der Maaten L, Hinton G. Visualizing data using t-SNE. J Mach Learn Res. 2008;9(2579–2605). Available from: http://jmlr.org/papers/v9/vandermaaten08a.html.

[34] Gautier EL, Shay T, Miller J, Greter M, Jakubzick C, Ivanov S (2012). Gene-expression profiles and transcriptional regulatory pathways that underlie the identity and diversity of mouse tissue macrophages. Nat Immunol..

[35] Palao T, Medzikovic L, Rippe C, Wanga S, Al-Mardini C, van Weert A, et al. Thrombospondin-4 mediates cardiovascular remodelling in angiotensin II-induced hypertension. Cardiovasc Pathol. 2018;35:12–9. Available from: https://pubmed.ncbi.nlm.nih.gov/29729633/. 10.1016/j.carpath.2018.03.003 29729633

[36] Steinhoff G, Nesteruk J, Wolfien M, Kundt G, Börgermann J, PERFECT Trial Investigators Group (2017). Cardiac function improvement and bone marrow response -: outcome analysis of the randomized PERFECT phase III clinical trial of intramyocardial CD133+ application after myocardial infarction. EBioMedicine..

[37] Shiba Y, Gomibuchi T, Seto T, Wada Y, Ichimura H, Tanaka Y (2016). Allogeneic transplantation of iPS cell-derived cardiomyocytes regenerates primate hearts. Nature..

[38] Heidt S, Roelen DL, Eijsink C, Eikmans M, Van Kooten C, Claas FHJ (2009). Calcineurin inhibitors affect B cell antibody responses indirectly by interfering with T cell help. Clin Exp Immunol..

[39] De Bruyne R, Bogaert D, De Ruyck N, Lambrecht BN, Van Winckel M, Gevaert P (2015). Calcineurin inhibitors dampen humoral immunity by acting directly on naive B cells. Clin Exp Immunol..

[40] Wallin EF, Hill DL, Linterman MA, Wood KJ. The Calcineurin inhibitor tacrolimus specifically suppresses human T follicular helper cells. Front Immunol. 2018;9. Available from: https://www.frontiersin.org/article/10.3389/fimmu.2018.01184/full. 10.3389/fimmu.2018.01184 PMC5990622 29904381

[41] Traitanon O, Mathew JM, La Monica G, Xu L, Mas V, Gallon L (2015). Differential effects of tacrolimus versus sirolimus on the proliferation, activation and differentiation of human B cells. Unutmaz D, editor. PLoS One..

[42] Carrigan KA, Saurer TB, Ijames SG, Lysle DT (2004). Buprenorphine produces naltrexone reversible alterations of immune status. Int Immunopharmacol..

[43] Filipczak-Bryniarska I, Nazimek K, Nowak B, Kozlowski M, Wąsik M, Bryniarski K (2018). In contrast to morphine, buprenorphine enhances macrophage-induced humoral immunity and as oxycodone, slightly suppresses the effector phase of cell-mediated immune response in mice. Int Immunopharmacol.

[44] Ribas JLC, da Silva CA, de Andrade L, Galvan GL, Cestari MM, Trindade ES (2014). Effects of anti-inflammatory drugs in primary kidney cell culture of a freshwater fish. Fish Shellfish Immunol..

[45] Krishnan V, Booker D, Cunningham G, Jadapalli JK, Kain V, Pullen AB (2019). Pretreatment of carprofen impaired initiation of inflammatory- and overlapping resolution response and promoted cardiorenal syndrome in heart failure. Life Sci..

[46] Mentkowski KI, Euscher LM, Patel A, Alevriadou BR, Lang JK (2020). Monocyte recruitment and fate specification after myocardial infarction. Am J Physiol Physiol..

[47] Tsou C-L, Peters W, Si Y, Slaymaker S, Aslanian AM, Weisberg SP (2007). Critical roles for CCR2 and MCP-3 in monocyte mobilization from bone marrow and recruitment to inflammatory sites. J Clin Invest..

[48] Deng K-Q, Li J, She Z-G, Gong J, Cheng W-L, Gong F-H (2017). Restoration of circulating MFGE8 (milk fat globule-EGF factor 8) attenuates cardiac hypertrophy through inhibition of Akt pathway. Hypertension..

[49] Wang B, Ge Z, Wu Y, Zha Y, Zhang X, Yan Y (2020). MFGE8 is down-regulated in cardiac fibrosis and attenuates endothelial-mesenchymal transition through Smad2/3-Snail signalling pathway. J Cell Mol Med..

[50] Nakaya M, Watari K, Tajima M, Nakaya T, Matsuda S, Ohara H (2016). Cardiac myofibroblast engulfment of dead cells facilitates recovery after myocardial infarction. J Clin Invest..

[51] Seok J, Warren HS, Cuenca AG, Mindrinos MN, Baker HV, Xu W (2013). Genomic responses in mouse models poorly mimic human inflammatory diseases. Proc Natl Acad Sci..

[52] Mestas J, Hughes CCW (2004). Of mice and not men: differences between mouse and human immunology. J Immunol..

[53] Payne KJ, Crooks GM (2007). Immune-cell lineage commitment: translation from mice to humans. Immunity..

[54] Rosshart SP, Herz J, Vassallo BG, Hunter A, Wall MK, Badger JH, et al. Laboratory mice born to wild mice have natural microbiota and model human immune responses. Science (80- ) [Internet]. 2019 Aug 2;365(6452). Available from: https://www.science.org/doi/10.1126/science.aaw4361. 10.1126/science.aaw4361 PMC7377314 31371577

