# Supplementary material for: CCR2 macrophage response determines the functional outcome following cardiomyocyte transplantation
Source: Genome Med. 2023 Aug 10;15:61. doi: 10.1186/s13073-023-01213-3 (PMC10416392; doi:10.1186/s13073-023-01213-3)
Supplement: Supplementary file 1 — Additional file 1: Supplement figures. Figure S1. Representative gating strategy for identifying the cardiac immune cells using flow cytometry and sorting them for single cell RNA sequencing. Figure S2. Single-cell RNA sequencing reveals the differentially expressed genes of the various cardiac immune cell clusters between C57BL/6J and Rag2del mice after MI. Figure S3. Assessment of cardiac ventricular remodelling. Figure S4. Differentially expressed transcripts in the heart and blood. Figure S5. GO terms for the DE transcripts in the heart and blood between Rag2delMI and Rag2delMI-CM groups. Figure S6. The most significant transcripts obtained using machine learning feature selection. [file 13073_2023_1213_MOESM1_ESM.zip › 13073_2023_1213_MOESM1_ESM/Additional file 1.docx]

**Supplementary figures**

**Figure S1. Representative gating strategy for identifying the cardiac immune cells using flow cytometry and sorting them for single cell RNA sequencing.** (A) Firstly, CD45 expressing cells were gated and only single cells were included into the analysis. Dead cells were excluded using Zombie-AmCyan dye. The viable CD45^+^ cells were further characterized based on the expression of various markers as following - *R1: CD11b^+^ cells (CD45^+^/CD11b^+^/CD11c^-^), R2: Dendritic cells (CD45^+^/CD11b^+^/CD11c^+^), R3: NK cells (CD45^+^/CD11b^-^/CD11c^-^/NK1.1^+^), R4: Monocytic cells (CD45^+^/CD11b^+^/CD11c^-^/Ly6G^lo^), R5: Neutrophils (CD45^+^/CD11b^+^/CD11c^-^/Ly6G^hi^), R6: Ly6C^lo^ or commonly known as M2 cells (CD45^+^/CD11b^+^/CD11c^-^/Ly6G^lo^/Ly6C^lo^), R7: Ly6C^hi^ or commonly known as M1 cells (CD45^+^/CD11b^+^/CD11c^-^/Ly6G^lo^/Ly6C^hi^), R8: CCR2^-^MHC-II^hi^ macrophages (CD45^+^/CD11b^+^/CD11c^-^/Ly6G^lo^/CCR2^-^/MHC-II^hi^), R9: CCR2^+^MHC-II^hi^ macrophages (CD45^+^/CD11b^+^/CD11c^-^/Ly6G^lo^/CCR2^+^/MHC-II^hi^), R10: Monocytes (CD45^+^/CD11b^+^/CD11c^-^/Ly6G^lo^/CCR2^+^/MHC-II^lo^), R11: CCR2^-^MHC-II^lo^ macrophages (CD45^+^/CD11b^+^/CD11c^-^/Ly6G^lo^/CCR2^-^/MHC-II^lo^)*. (B) Representative gating strategy to isolate immune cells from the heart for single-cell RNA sequencing analysis. Briefly, mouse hearts were isolated seven days after MI and enzymatically digested. CD45 cells were then enriched using magnetic beads. Viable and non-apoptotic R1: macrophages/monocytes (CD45^+^CD11b^+^CD11c^-^DAPI^-^Lactadherin^lo^), R2: dendritic cells (CD45^+^CD11b^+^CD11c^+^ DAPI^-^Lactadherin^lo^) and R3: NK cells (CD45^+^CD11b^-^CD11c^+^NK1.1^+^ DAPI^-^Lactadherin^lo^) were then sorted and pooled.

**Figure S2. Single-cell RNA sequencing reveals the differentially expressed genes of the various cardiac immune cell clusters between C57BL/6J and Rag2^del^ mice after MI.** (A) Top DE genes in the different cardiac immune cell clusters between C57BL/6J and Rag2^del^ mice. (B) Pathway enrichment analysis of the DE genes between the various macrophage clusters and (C) CCR2^+^MHC-II^hi^ macrophages between C57BL/6J and Rag2^del^ mice using WikiPathways.

**Figure S3. Assessment of cardiac ventricular remodelling.** (A) Assessment of fibrotic area in the heart four weeks after MI using Fast Green/Sirius Red staining (n=5-7). The heart slices were observed using a 10x objective. The relative fibrotic area of the four segments – lateral, septal, inferior and anterior was then analyzed using ImageJ. (B) Assessment of CD31^+^ cells in the remote area of the heart four weeks after MI (n=5-7). CD31^+^ cells are stained red and the nuclei are stained blue using DAPI. Scale bar represents 20 µm. ‘MI’ refers to infarct group, ‘MI-CM’ refers to cell transplanted group Values are represented as Mean±SEM.

**Figure S4. Differentially expressed transcripts in the heart and blood.** Rag2^del^ mice were subjected to MI with or without cardiomyocyte transplantation and seven days after MI, the RNA from the heart and blood was isolated and analysed via a ClariomTM D microarray. Heatmap of annotated DE transcripts found between Rag2^del^MI and Rag2^del^MI-CM in the heart (A) and blood (B). ‘MI’ refers to infarct group, ‘MI-CM’ refers to cell transplanted group. Transcripts are considered DE, if they show a two-fold change and have a p-value <0.05. Upregulated targets are coloured red while the downregulated targets are coloured green.

**Figure S5. GO terms for the DE transcripts in the heart and blood between Rag2^del^MI and Rag2^del^MI-CM groups.** GO enrichment analysis of the DE transcripts between Rag2^del^MI and Rag2^del^MI-CM groups with the GO terms, their respective GO ID and the associated transcripts found in the blood and heart. The analysis was performed using ClueGO. ‘MI’ refers to infarct group, ‘MI-CM’ refers to cell transplanted group.

**Figure S6. The most significant transcripts obtained using machine learning feature selection.** A machine learning (ML) based feature selection approach was applied to independently rank the importance of the identified significantly DE transcripts. (A) The resulting ML model was cross-validated and received a very good 91.6 % AUC prediction performance. The 20 important DE transcripts between cell treated mice and untreated mice were identified using machine learning algorithms (RF) with the following ROC curve metrics in the heart. (B) The fold change in gene expression normalized to the BL6MI group values of the genes marked in gold from *Figure 6* in the heart are illustrated as a box plot with the median, upper and lower limits. Significance was calculated using ANOVA with Bonferroni post-hoc correction. *- p<0.05. (C) Circos plots illustrating the conserved regions of identified transcripts, Gm24643 and TC0600000166 in humans. ‘MI’ refers to infarct group, ‘MI-CM’ refers to cell transplanted group.
