# Supplementary figures and images for: CCR2 macrophage response determines the functional outcome following cardiomyocyte transplantation
Source: Genome Med. 2023 Aug 10;15:61. doi: 10.1186/s13073-023-01213-3 (PMC10416392; doi:10.1186/s13073-023-01213-3)

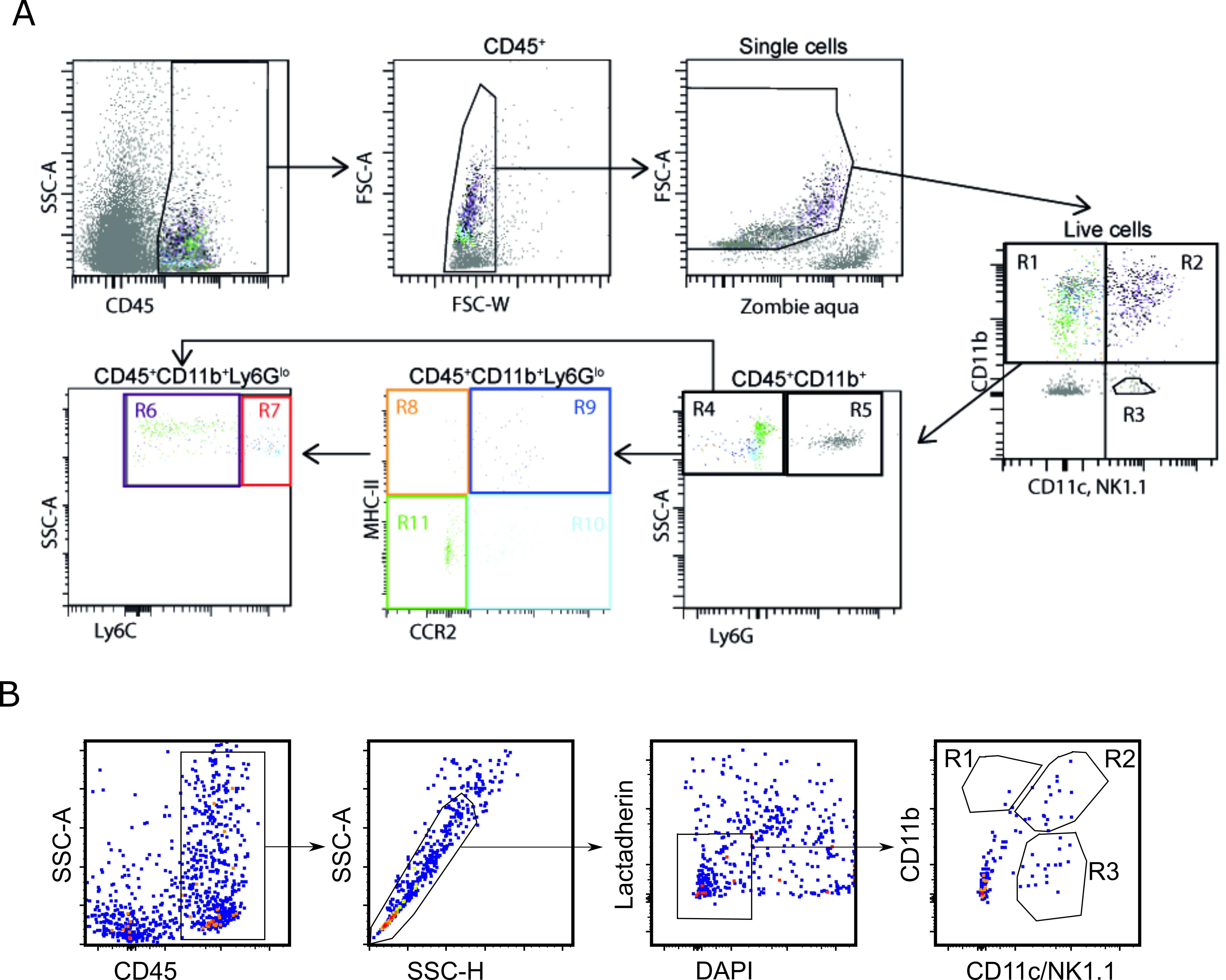

Supplement: Supplementary file 1 — Additional file 1: Supplement figures. Figure S1. Representative gating strategy for identifying the cardiac immune cells using flow cytometry and sorting them for single cell RNA sequencing. Figure S2. Single-cell RNA sequencing reveals the differentially expressed genes of the various cardiac immune cell clusters between C57BL/6J and Rag2del mice after MI. Figure S3. Assessment of cardiac ventricular remodelling. Figure S4. Differentially expressed transcripts in the heart and blood. Figure S5. GO terms for the DE transcripts in the heart and blood between Rag2delMI and Rag2delMI-CM groups. Figure S6. The most significant transcripts obtained using machine learning feature selection. [file 13073_2023_1213_MOESM1_ESM.zip › 13073_2023_1213_MOESM1_ESM/Additional file 1 - Fig S1.png]

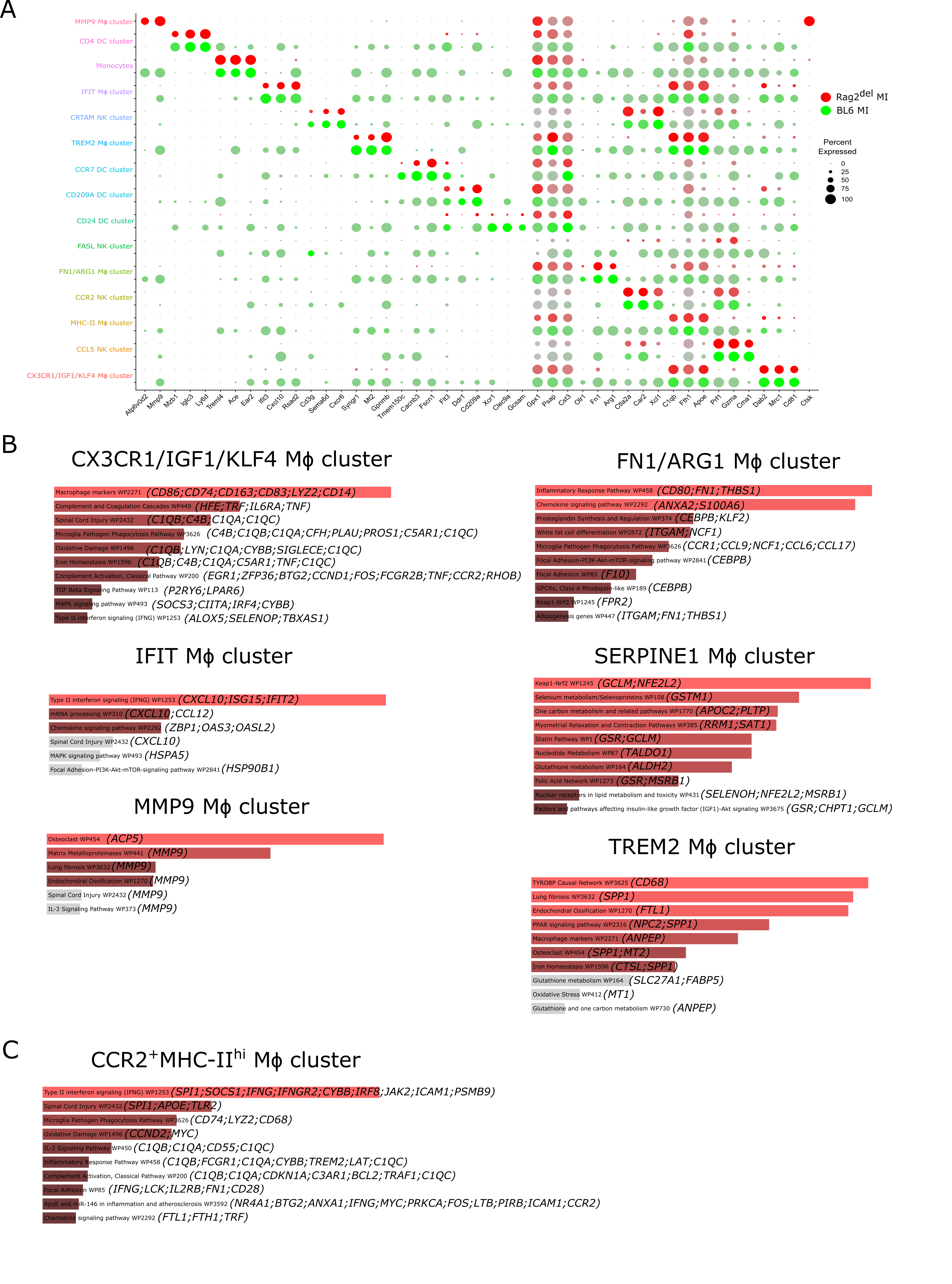

Supplement: Supplementary file 1 — Additional file 1: Supplement figures. Figure S1. Representative gating strategy for identifying the cardiac immune cells using flow cytometry and sorting them for single cell RNA sequencing. Figure S2. Single-cell RNA sequencing reveals the differentially expressed genes of the various cardiac immune cell clusters between C57BL/6J and Rag2del mice after MI. Figure S3. Assessment of cardiac ventricular remodelling. Figure S4. Differentially expressed transcripts in the heart and blood. Figure S5. GO terms for the DE transcripts in the heart and blood between Rag2delMI and Rag2delMI-CM groups. Figure S6. The most significant transcripts obtained using machine learning feature selection. [file 13073_2023_1213_MOESM1_ESM.zip › 13073_2023_1213_MOESM1_ESM/Additional file 1 - Fig S2.png]

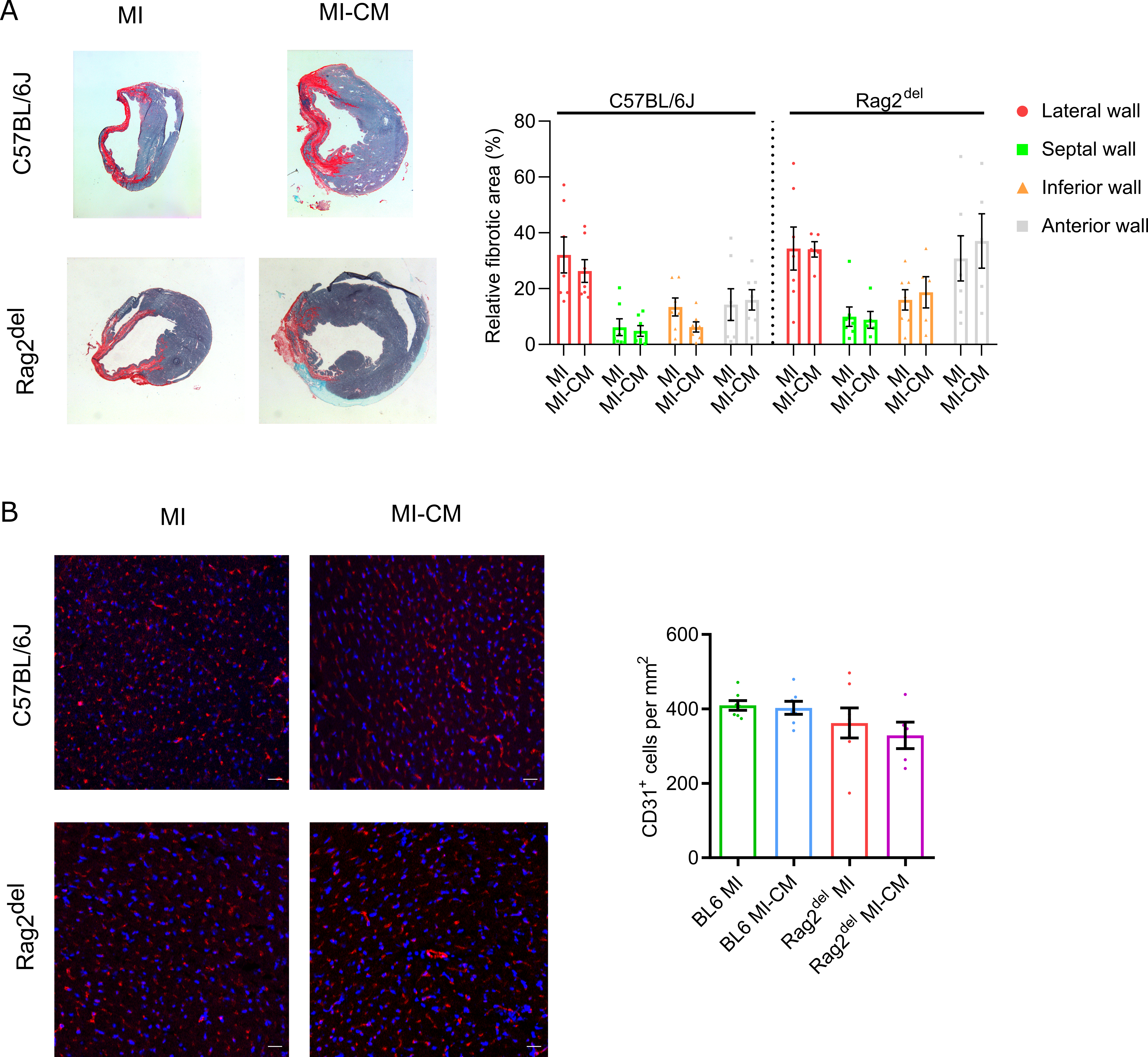

Supplement: Supplementary file 1 — Additional file 1: Supplement figures. Figure S1. Representative gating strategy for identifying the cardiac immune cells using flow cytometry and sorting them for single cell RNA sequencing. Figure S2. Single-cell RNA sequencing reveals the differentially expressed genes of the various cardiac immune cell clusters between C57BL/6J and Rag2del mice after MI. Figure S3. Assessment of cardiac ventricular remodelling. Figure S4. Differentially expressed transcripts in the heart and blood. Figure S5. GO terms for the DE transcripts in the heart and blood between Rag2delMI and Rag2delMI-CM groups. Figure S6. The most significant transcripts obtained using machine learning feature selection. [file 13073_2023_1213_MOESM1_ESM.zip › 13073_2023_1213_MOESM1_ESM/Additional file 1 - Fig S3.png]

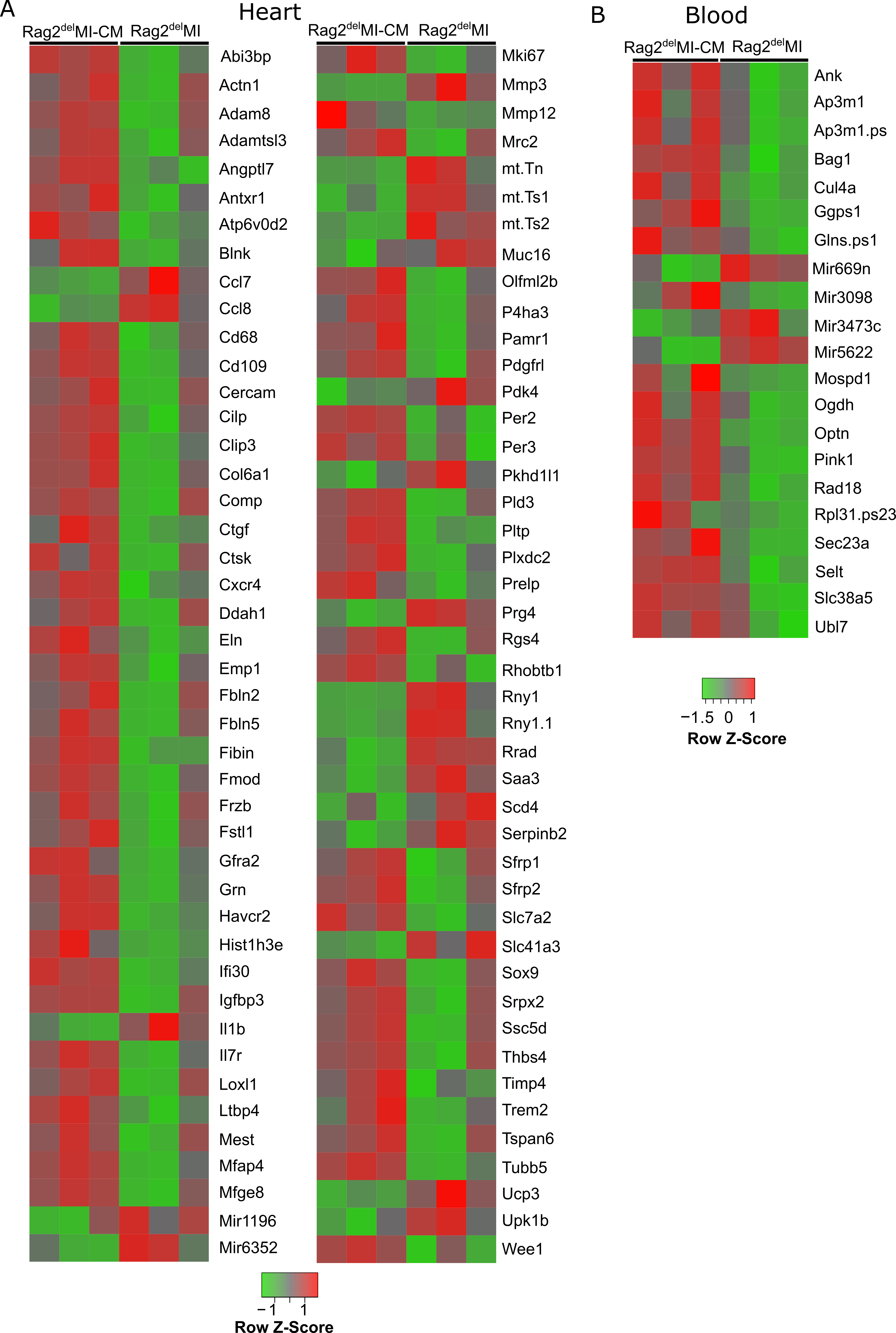

Supplement: Supplementary file 1 — Additional file 1: Supplement figures. Figure S1. Representative gating strategy for identifying the cardiac immune cells using flow cytometry and sorting them for single cell RNA sequencing. Figure S2. Single-cell RNA sequencing reveals the differentially expressed genes of the various cardiac immune cell clusters between C57BL/6J and Rag2del mice after MI. Figure S3. Assessment of cardiac ventricular remodelling. Figure S4. Differentially expressed transcripts in the heart and blood. Figure S5. GO terms for the DE transcripts in the heart and blood between Rag2delMI and Rag2delMI-CM groups. Figure S6. The most significant transcripts obtained using machine learning feature selection. [file 13073_2023_1213_MOESM1_ESM.zip › 13073_2023_1213_MOESM1_ESM/Additional file 1 - Fig S4.png]

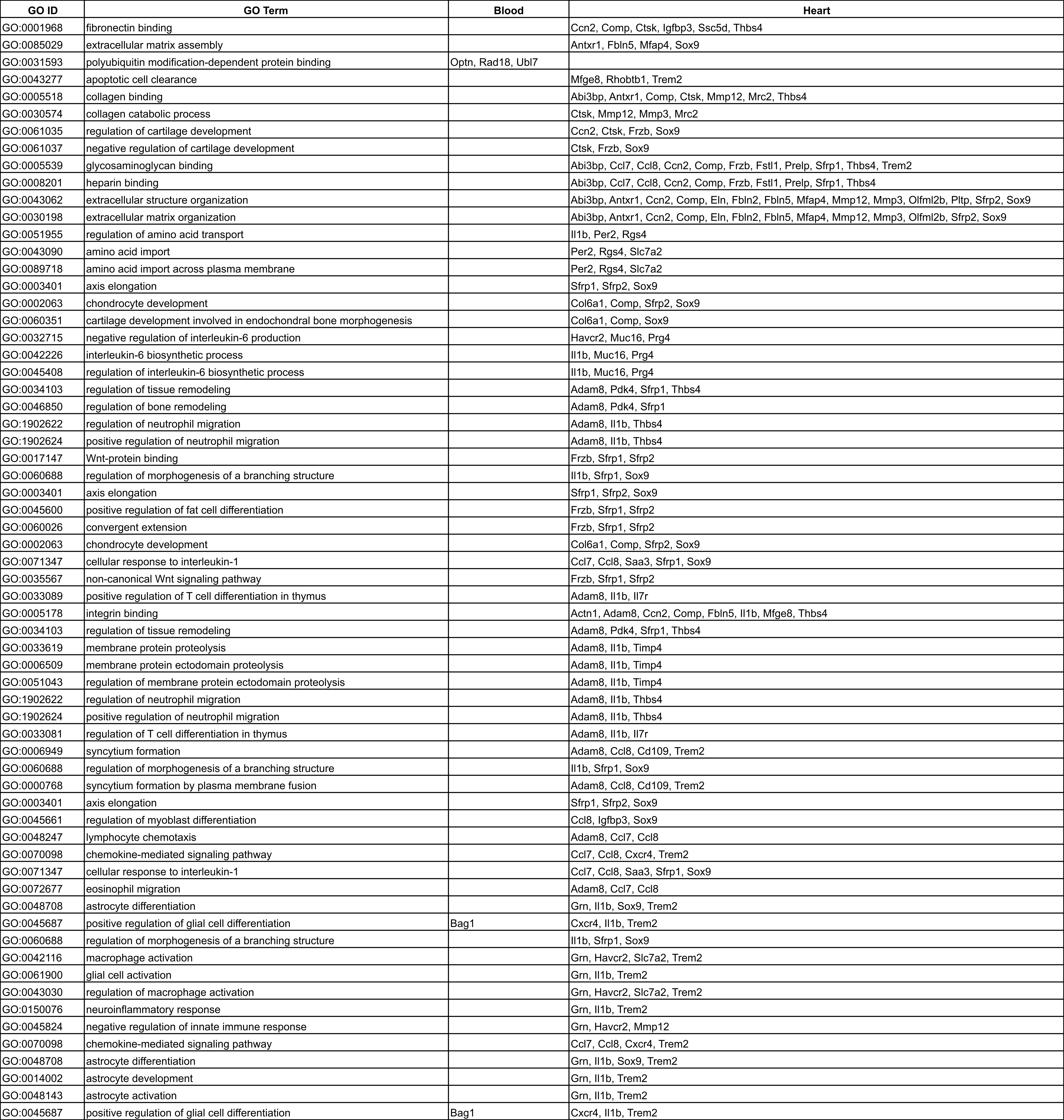

Supplement: Supplementary file 1 — Additional file 1: Supplement figures. Figure S1. Representative gating strategy for identifying the cardiac immune cells using flow cytometry and sorting them for single cell RNA sequencing. Figure S2. Single-cell RNA sequencing reveals the differentially expressed genes of the various cardiac immune cell clusters between C57BL/6J and Rag2del mice after MI. Figure S3. Assessment of cardiac ventricular remodelling. Figure S4. Differentially expressed transcripts in the heart and blood. Figure S5. GO terms for the DE transcripts in the heart and blood between Rag2delMI and Rag2delMI-CM groups. Figure S6. The most significant transcripts obtained using machine learning feature selection. [file 13073_2023_1213_MOESM1_ESM.zip › 13073_2023_1213_MOESM1_ESM/Additional file 1 - Fig S5.png]

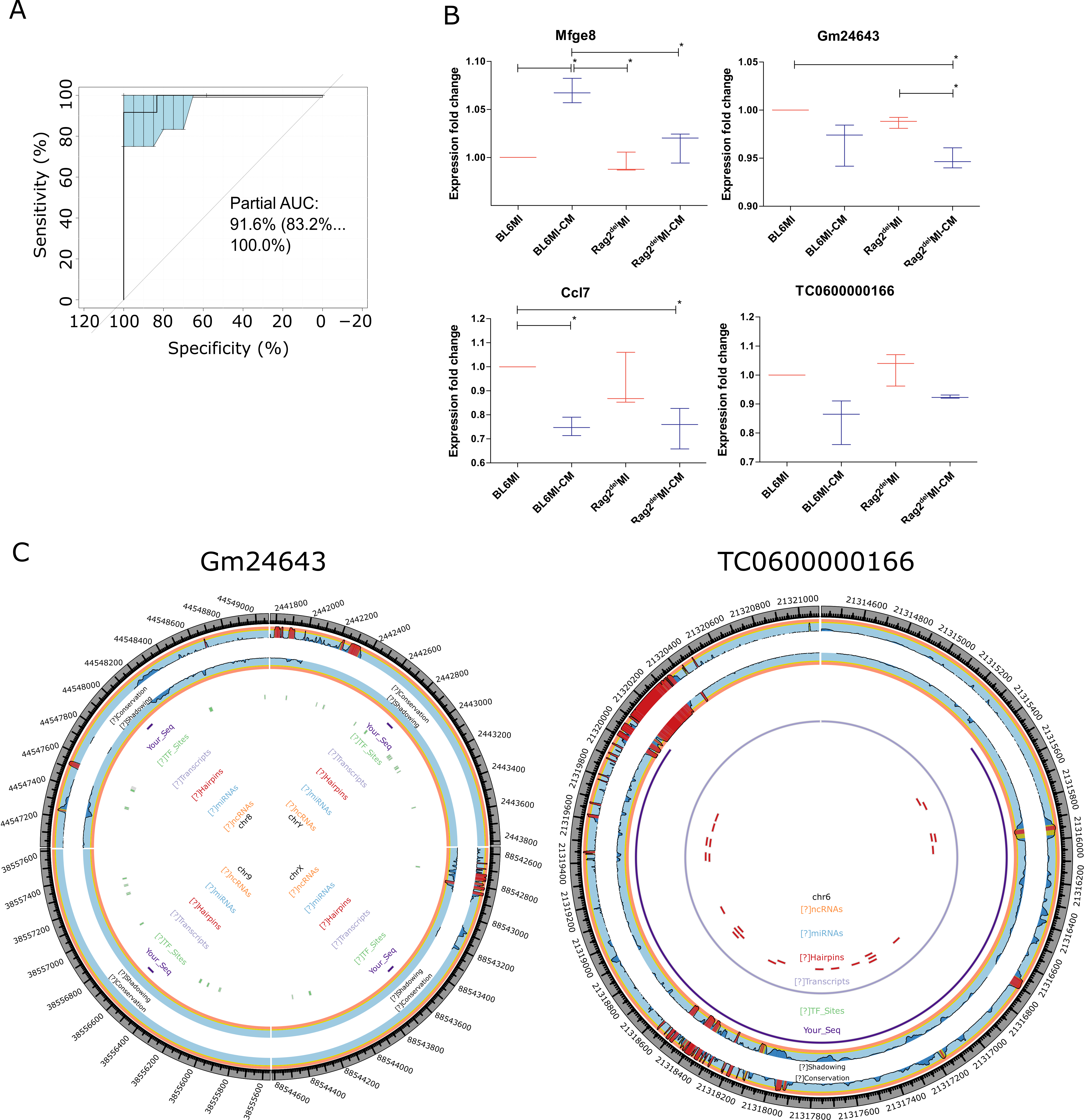

Supplement: Supplementary file 1 — Additional file 1: Supplement figures. Figure S1. Representative gating strategy for identifying the cardiac immune cells using flow cytometry and sorting them for single cell RNA sequencing. Figure S2. Single-cell RNA sequencing reveals the differentially expressed genes of the various cardiac immune cell clusters between C57BL/6J and Rag2del mice after MI. Figure S3. Assessment of cardiac ventricular remodelling. Figure S4. Differentially expressed transcripts in the heart and blood. Figure S5. GO terms for the DE transcripts in the heart and blood between Rag2delMI and Rag2delMI-CM groups. Figure S6. The most significant transcripts obtained using machine learning feature selection. [file 13073_2023_1213_MOESM1_ESM.zip › 13073_2023_1213_MOESM1_ESM/Additional file 1 - Fig S6.png]
